# Metaproteomic analysis decodes trophic interactions of microorganisms in the dark ocean

**DOI:** 10.1038/s41467-024-50867-z

**Published:** 2024-07-30

**Authors:** Zihao Zhao, Chie Amano, Thomas Reinthaler, Federico Baltar, Mónica V. Orellana, Gerhard J. Herndl

**Affiliations:** 1 Department of Functional and Evolutionary Ecology, Bio-Oceanography and Marine Biology Unit, University of Vienna, Vienna, Austria; 2 Shanghai Engineering Research Center of Hadal Science and Technology, College of Marine Sciences, Shanghai Ocean University, Shanghai, China; 3 Polar Science Center, Applied Physics Laboratory, University of Washington, Seattle, WA USA; 4 Institute for Systems Biology, Seattle, WA USA; 5 NIOZ, Department of Marine Microbiology and Biogeochemistry, Royal Netherlands Institute for Sea Research, Utrecht University, Den Burg, The Netherlands; 6 Environmental & Climate Research Hub, University of Vienna, Vienna, Austria

**Keywords:** Microbial biooceanography, Microbial biooceanography, Marine biology, Microbial ecology

## Abstract

Proteins in the open ocean represent a significant source of organic matter, and their profiles reflect the metabolic activities of marine microorganisms. Here, by analyzing metaproteomic samples collected from the Pacific, Atlantic and Southern Ocean, we reveal size-fractionated patterns of the structure and function of the marine microbiota protein pool in the water column, particularly in the dark ocean (>200 m). Zooplankton proteins contributed three times more than algal proteins to the deep-sea community metaproteome. Gammaproteobacteria exhibited high metabolic activity in the deep-sea, contributing up to 30% of bacterial proteins. Close virus-host interactions of this taxon might explain the dominance of gammaproteobacterial proteins in the dissolved fraction. A high urease expression in nitrifiers suggested links between their dark carbon fixation and zooplankton urea production. In summary, our results uncover the taxonomic contribution of the microbiota to the oceanic protein pool, revealing protein fluxes from particles to the dissolved organic matter pool.

## Abstract (web summary)

This study analysed metaproteomic samples collected from the Pacific, Atlantic and Southern Oceans to reveal size-fractionated patterns of the structure and function of the marine microbiota protein pool in the water column.

## Introduction

Microbial utilization of organic matter is one of the drivers regulating the marine carbon cycle^1^. Most of the marine organic matter is biosynthesized in the surface ocean, and about 20% is exported to the ocean’s interior via various mechanisms^2,3^. Particulate organic matter (POM) has been proposed as the main trophic basis for heterotrophic microbes in the deep-sea because the bioavailability of the bulk of dissolved organic matter (DOM) is low for deep-sea microbes^4–6^. The POM flux driven by zooplankton is proposed to become prominent in the deep ocean as most sinking algal material is consumed within the top 1000 m^7–9^. Additionally, zooplankton can release large quantities of urea^10,11^, which serves as an additional ammonia source supporting nitrification-mediated dissolved inorganic carbon (DIC) fixation in the aphotic zone^12^. Thus, zooplankton activity and their release of urea increases organic matter availability in the meso- and bathypelagic ocean and sustains the metabolic activity of deep-sea microbes^13–15^. Gammaproteobacteria are the main producers of extracellular enzymes^16^. Even in the deep ocean, they can hydrolyze POM where their metabolic activity is comparable to other taxonomic groups despite being impacted by high hydrostatic pressure conditions^16,17^. Yet, the relatively low abundance of Gammaproteobacteria based on 16S rRNA genes^18^ appears to be in contrast with their activity and might be indicative of significant cell losses due to viral lysis of this bacterial group^19^.

All these findings indicate potentially intrinsic links between zooplankton, bacteria, archaea, and viruses in the carbon cycling process. However, evidence supporting these connections remains poor. Specifically, the relative contribution of zooplankton and algae to the POM pool throughout the oceanic water column remains unclear. Whether urea facilitates DIC fixation in the global ocean is also not well documented, as is the role of Gammaproteobacteria in POM remineralization and their interaction with viruses.

We characterized the structure and function of marine microbial communities using a metaproteomics approach to assess the relative abundance of proteins in individual taxa. We also focused on microbial enzyme expression profiles to determine the links between organic matter supply and microbial activities. Proteins are essential biomolecules for microorganisms but also significant sources of organic matter. While the protein expression level is a direct response to the (micro)environment^20–22^, the abundance of proteins is a measure of the contribution of individual populations to the total biomass in a specific depth layer of the oceanic water column^23^. Despite the relatively low resolution of metaproteomics compared to metagenomic and metatranscriptomic analysis, the non-amplification nature of mass spectrometry analysis supports comparison at the protein level across superkingdoms. It produces semi-quantitative results on the contribution of each taxon to the total protein pool^23^.

In this work, we show that the taxonomic origin and the expression pattern of the marine microbial protein pool is size fractionated. The differences between size fractions highlight the essential role of microbial activities in mediating the flux of organic matter from the POM pool to the DOM pool.

## Results and discussion

### Protein profiles of the marine plankton community

**Fig. 1:**
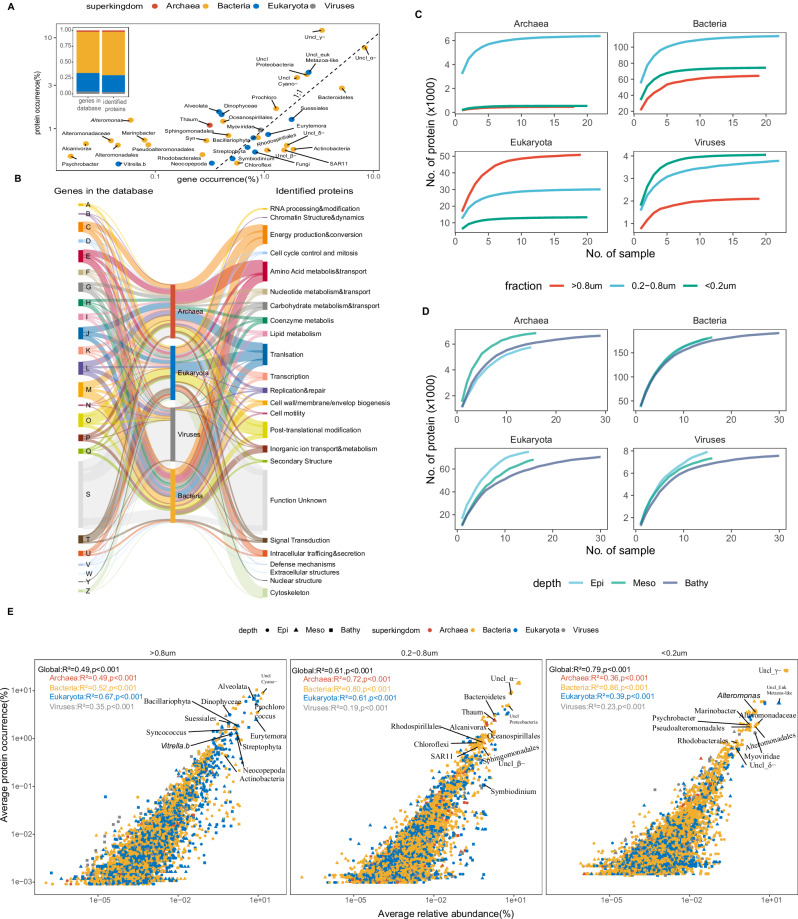
Protein sequences identified in the metaproteome. **A** Relationship between protein occurrence and the occurrence of corresponding genes in the database. The protein/gene sequences were grouped according to NCBI taxonomy IDs. The dashed line represents the 1:1 ratio. The inserted bar-plot shows the taxonomic composition in the gene database and total identified proteins at superkingdom level. **B** Comparison between gene occurrence and protein occurrence according to clusters of orthologous groups (COGs). **C**–**D** Rarefaction curve of detected proteins from different size-fractions and depth. **E** Relationship (two-sided Spearman’s rank correlation coefficient) between protein occurrence and protein relative abundance in the metaproteome dataset. Protein sequences were grouped according to NCBI taxonomy IDs. Each datapoint represents the mean value of relative abundance (*x*-axis) and protein occurrence (*y-*axis) from the same depth layer in each fraction. Taxonomic labels are given to the datapoint where the highest mean relative abundance was detected. Uncl, unclassified; α-, alphaproteobacteria; β-, betaproteobacteria; γ-, gammaproteobacteria; δ-, deltaproteobacteria; Source data are provided as a Source Data file.

We collected 61 metaproteomics samples from 22 stations between 5 m and 4000 m depth from the major ocean basins and three size-fractions (>0.8 μm, 0.2–0.8 μm, and <0.2 μm, 48 samples from 16 stations covered all three size-fractions) to cover eukaryotes, bacteria, archaea, and viruses (Figs. 1A, S1A–C, Supplementary Data 1, see Methods). Data-dependent metaproteomic analysis heavily relies on the completeness of the sequence database for spectral identification. Thus, a well-curated gene catalog from global scale metagenomic and metatranscriptomic surveys^24–27^ significantly improves the metaproteomic protein identification and functional profiling. We employed an optimized database construction strategy for robust protein identification^28^. Metagenomic assemblies from the same sampling stations were combined with publicly available metagenomics/metatranscriptomics assemblies to obtain a deep coverage of microorganisms in the ocean (including micro-eukaryotes, bacteria, archaea, and viruses) throughout the entire water column from global ocean expeditions^24–27^. A two-step search approach was implemented to minimize the high false discovery rate in protein identification caused by a large database^29^. Searching against such a comprehensive database covering marine micro-eukaryotes, prokaryotes, and viruses (Table S1), we identified 234,550 protein entries (Table S2, see Methods). Among them, 90% were bacterial (156,187) and eukaryotic (55,494) sequences, while archaeal (7163) and viral (6213) sequences contributed less than 10% (Table S3). The taxonomic occurrence of identified proteins at the superkingdom level was similar to the corresponding genes in the database (Fig. 1A). A clear difference was found, however, when grouping the proteins/genes according to the lowest taxonomic linkages (taxonomic IDs, Fig. 1A). While a linear relationship was found between the protein and the corresponding gene occurrence in the database, protein sequences affiliated to Alteromonadales (including Alteromonadaceae and *Alteromonas*) showed high occurrence (~1%) in the metaproteome. Still the occurrence of corresponding genes in the database was low (<0.1%). (Fig. 1A). In the gene database derived from metagenomic and metatranscriptomic analyses, less than 50% of predicted genes were taxonomically and functionally classified^24,25,30^. In the metaproteome, however, more than 70% of the identified protein sequences (ca. 150,000 protein sequences) were taxonomically classified at the class level and functionally annotated in at least one functional database (Fig. S1D, E), which is similar to other metaproteomic results covering both micro-eukaryotes and prokaryotes^31^. The function of identified proteins in the metaproteome based on the Cluster of Orthologous Groups (COG) was also distinct from corresponding genes in the database (Fig. 1B). Proteins involved in amino acid metabolism had a high occurrence in bacteria and archaea, and proteins involved in intracellular trafficking, secretion, and cytoskeleton interlinking showed high occurrence in eukaryotes. A clear size-fractionated pattern in sequence richness was observed between superkingdoms (Fig. 1C), where eukaryotic proteins were rich in the >0.8 µm size fraction, bacterial and archaeal proteins dominated the 0.2–0.8 µm fraction. In comparison viral proteins were mainly found in the <0.2 µm fraction. The depth stratification was also profound for eukaryotes and archaea, while the richness of bacterial proteins did not change with depth (Fig. 1D). Previous reports show that proteins occurrence in marine microorganisms (micro-eukaryotes, prokaryotes and virus) varies across different size-fractions (particulate, free-living and dissolved fractions)^32–35^. By comparing protein occurrence with the relative abundance of proteins, we found strong correlations between protein occurrence and protein relative abundance (Fig. 1E, *R*^2^ > = 0.49, *p* < 0.001). The major contributors to the marine protein pool varied across size fractions. Proteins from cyanobacteria, eukaryotic algae, and zooplankton (copepods) exhibited a high occurrence and relative abundance in the >0.8 µm fraction. Heterotrophic bacterial proteins showed high occurrence and relative abundance in the <0.8 µm (0.2–0.8 µm and <0.2 µm) fractions with taxonomic variations. For example, proteins from SAR11, Chloroflexi, and Bacteroidetes showed high occurrence and relative abundance in the 0.2–0.8 µm fraction but the occurrence and relative abundance of proteins from Alteromonadales were high in the <0.2 µm fraction. Proteins from prokaryotic autotrophs such as Thaumarchaea also exhibited a high occurrence and relative abundance in the 0.2–0.8 µm fraction. However, protein sequences related to Myoviridae were the most abundant (both occurrence and relative abundance) viral proteins in the <0.2 µm fraction (Fig. 1E).

**Fig. 2:**
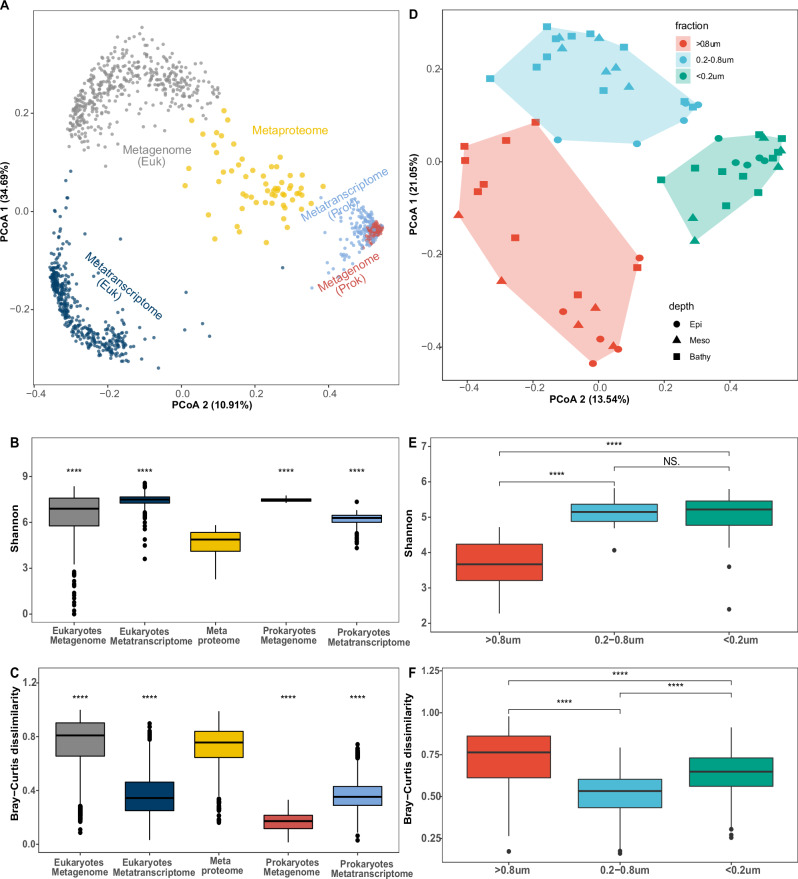
Diversity profiles of the microbial metaproteome. **A** Principal coordinate analysis of KO based functional profiles obtained from the metaproteome, metagenome, and metatranscriptome of the planktonic community. Euk, samples enriched in micro-eukaryotes (samples collected in the >0.8 µm fraction), Prok, samples enriched in prokaryotes (samples collected in the 0.2–3 µm fraction). Diversity comparison shows that the metaproteome (*n* = 61) has low α-diversity (**B**) but high β-diversity (**C**) in comparison to the metagenomics (eukaryotic metagenome, *n* = 158; prokaryotic metagenome, *n* = 345) and –transcriptomics (eukaryotic metatranscriptome, *n* = 157; prokaryotic metatranscriptome, *n* = 521) dataset; significance test (Wilcoxon test) was made using the metaproteome as reference. **D** Principal coordinate analysis of KO based functional profiles of metaproteomes between depths and size-fractions. Size-fraction based clustering patterns are observed (Permanova, *p* < 0.05). Metaproteomic samples collected from the >0.8 μm (*n* = 19) fraction shows low α-diversity (**E**) but high β-diversity (**F)** than samples collected from the 0.2–0.8 μm (*n* = 22) and <0.2 μm (*n* = 20) fractions; Box shows median and interquartile range (IQR); whiskers show 1.5 × IQR of the lower and upper quartiles or range; outliers extend to the data range. Statistics are based on Wilcoxon test (two-side). ****p < 0.0001, ns, not significant. Source data are provided as a Source Data file.

We further performed a functional assessment of the metaproteome using KEGG-orthologue (KO) based analysis^36^. Protein sequences were grouped into 3817 KOs (Supplementary Data 3). Compared to the metagenomic and metatranscriptomic analysis, the total number of KOs was relatively low in our metaproteome (Fig. S2A, B), partially due to the limited sample size, and the resolution of mass spectrometry. However, the relative abundance of KOs revealed that KOs with high relative abundances in the metagenome and metatranscriptome were also highly abundant in the metaproteome (Fig. S2C, D). The functional composition (based on the relative abundance of KOs) of the metaproteome, however, was significantly different (Figs. 2A, S2C, PERMANOVA, *p* < 0.05) from the metagenome and metatranscriptome for both, the eukaryotic-enriched (>0.8 µm) and prokaryotic-enriched (0.2–3 µm) community^24,25,30^. Diversity analysis on KOs showed that the metaproteomics dataset had the lowest alpha diversity but a high beta diversity (Wilcoxon test, *p* < 0.05, Fig. 2B, C). Clusters of size fractions were found in the metagenomic and –transcriptomic dataset (Fig. 2A), as well as at the metaproteome level (Fig. 2D, PERMANOVA, *p* < 0.05). However, the metaproteomic samples were collected from disparate ocean regions (Fig. S1A). The metaproteome KO profile of the >0.8 μm size-fraction exhibited the lowest alpha diversity but the highest variance (Bray-Curtis dissimilarity, Fig. 2E, F). In contrast, in the <0.8 μm size-fraction (both 0.2–0.8 μm and <0.2 μm), alpha-diversity was highest and variance lowest (Wilcoxon test, *p* < 0.05, Fig. 2E, F). These results suggest that the high variance in the metaproteome is driven by the KO profile of the >0.8 μm size-fraction where low within-site diversity of proteins but high diversity across sites were observed (Fig. 2E, F). As the samples were collected from diverse sites (Fig. S1A), differences in epipelagic biogeochemistry might have affected the phytoplankton community and shaped the diversity of particles^37^, which might have led to the high beta-diversity in the >0.8 μm size-fraction. A similar pattern was also found for the prokaryotic metatranscriptomes (Fig. 2B, C). The changes in the protein profiles between samples (Fig. 2B, C) imply that the genome (particularly prokaryotic genome) responds to the environment by transcribing and translating proteins/enzymes adapted to specific functions with adequate expression levels^20,38^. This observation is consistent with the fact that while the protein-coding gene abundance reflects long-term adaptive mechanism of microbial colonization between depths or size-fractions^26,39^, the transcription response^25^ and translational regulation of protein synthesis^20^ control the near-instantaneous microbial interaction with the environment such as particles^38,40^.

To determine the key enzymes/proteins in the different size fractions, we identified 1630 (40% of total KOs) KOs where the relative abundance was significantly different either among size-fractions (1387 KOs) or depth-strata (412 KOs) (Wilcoxon test, *p* < 0.05, Figs. S3, 4, Supplementary Data 4). There were 178 KOs differentially abundant in both, depth layers and size-fractions, predominately originating from eukaryotes (ca. 60%, pie chart in Fig. S3A). However, bacteria dominated the differentially abundant unique KOs in size-fractions (ca. 70%) and depth (55%) (pie chart in Fig. S3A). The highest number of differentially abundant KOs was found between the <0.2 μm and 0.2–0.8 μm size-fraction, and the difference between epi- and bathypelagic was substantial (Fig. S3B–D). In contrast, the number of differentially abundant KOs was relatively low between the <0.2 μm and >0.8 μm fraction (Fig. S3B). This KO distribution suggests that the bacterial proteins in the <0.2 μm fraction likely originate from the particle-attached community retained on the 0.8 μm pore-size filters.

**Fig. 3:**
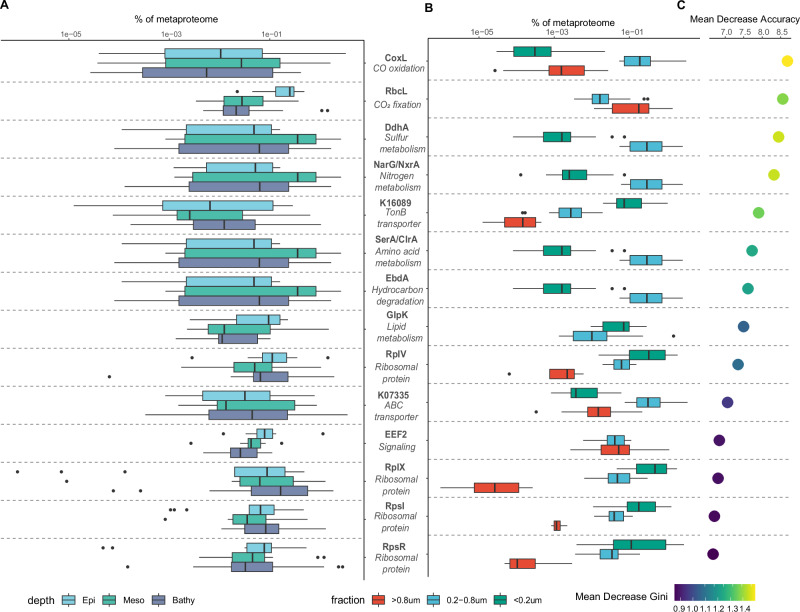
Relative abundance profiles and variable importance of the top 14 proteins/enzymes identified using random forest classification. These 14 KOs were identified as feature KOs distinguishing KO profiles in metaproteomes collected from different size-fractions. The boxplot shows the relative abundance of these 14 KOs in different depth layers (**A**) and size-fractions (**B**). The importance of the 14 KOs in the different size-fractions predicted by random forest analysis is shown in the dot plot (**C**). The Mean Decrease Accuracy shows how much accuracy the model losses by excluding each variable and the mean decrease in Gini coefficient reflects how each variable contributes to the homogeneity of the nodes and leaves in the random forest result. Box shows median and interquartile range (IQR); whiskers show 1.5 × IQR of the lower and upper quartiles or range; outliers extend to the data range. Epi, samples collected from epipelagic (<200 m, *n* = 15); Meso, samples collected from mesopelagic (200–1000 m, *n* = 16); Bathy, samples collected from bathypelagic (>1000 m, *n* = 30); >0.8 μm, samples collected from the >0.8 μm fraction (*n* = 19); 0.2–0.8 μm, samples collected from the 0.2–0.8 μm fraction (*n* = 22); <0.2 μm, samples collected from the <0.2 μm fraction (*n* = 20). Source data are provided as a Source Data file.

Functional annotations of microbial protein profiles revealed that photosynthesis, nitrification/denitrification, microbial chemotaxis, and motility exhibited different expression profiles between size-fractions (Fig. S4, Supplementary Data 4). Fourteen KOs were predicted to be responsible for the functional clustering between the size-fractions using a machine-learning random forest^41^ classification (Figs. 3, S5). Among these KOs were enzymes involved in C1 metabolism (carbon-monoxide dehydrogenase, CoxL), CO_2_ fixation (Rubisco, RbcL), nitrification/denitrification (nitrate reductase/nitrite oxidoreductase, NarG/NxrA), sulfur metabolism (dimethylsulfide dehydrogenase, DdhA) and transporter proteins, all different in relative abundance in the different size-fractions (Fig. 3B, C). Only enzymes related to photosynthesis (RbcL) were depth-related (Fig. 3A), consistent with the dominance of phytoplankton in the euphotic zone. It is also noticeable that ribosomal proteins (RplV, RplX, RpsI, RpsR) exhibited their highest relative abundance in the <0.2 µm fraction. Ribosomal proteins are cytoplasmic proteins mediating protein synthesis inside cells. High expression levels of transcripts encoding ribosomal proteins were reported during viral infection processes because viruses employ a cellular metabolism for viral protein synthesis^42^. The high relative abundance of ribosomal proteins found in the <0.2 µm fraction suggests infected bacterial cells exhibit a high translation activity (probably due to viral replication) and release cellular components (including ribosomal proteins) into the ambient water after cell lysis.

### Zooplankton-supported deep-sea particulate protein flux

**Fig. 4:**
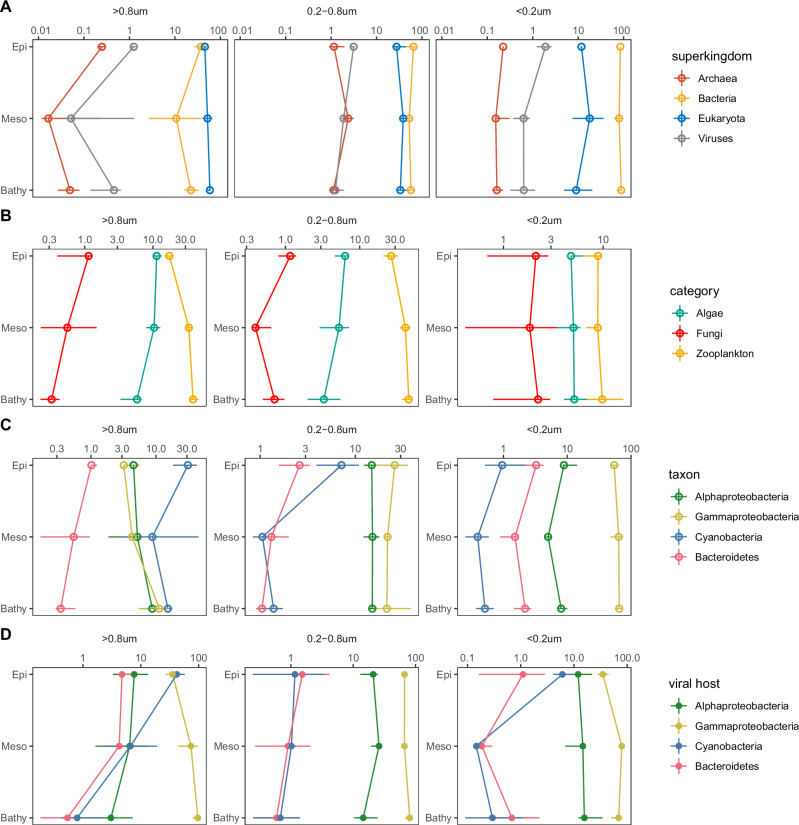
Changes in taxonomic composition of the eukaryotic, bacterial, and viral community in the metaproteome dataset. **A** Relative abundance of proteins of Archaea, Bacteria, Eukaryotes and Viruses in each size-fraction. **B** Relative abundance of proteins of algae, zooplankton, and fungi in each size-fraction. **C** Relative abundance of proteins of major bacterial groups in each size-fraction. **D** Relative abundance of proteins of viruses infecting different bacterial hosts in each size-fraction. The points and ranges show the medians, the 25th and 75th IQR. Epi, samples collected from epipelagic (<200 m, *n* = 15); Meso, samples collected from mesopelagic (200–1000 m, *n* = 16); Bathy, samples collected from bathypelagic (>1000 m, *n* = 30); >0.8 μm, samples collected from the >0.8 μm fraction (*n* = 19); 0.2–0.8 μm, samples collected from the 0.2–0.8 μm fraction (*n* = 22); <0.2 μm, samples collected from the <0.2 μm fraction (*n* = 20). Source data are provided as a Source Data file.

The taxonomic composition of the metaproteome also exhibited a size-clustering pattern (Fig. 4A, Supplementary Data 5). The relative abundance of eukaryotic and bacterial proteins constituted 70–80% to the total proteome in our metaproteomic dataset with varying contributions among the size-fractions (Fig. 4A). In the >0.8 μm size-fraction, the ratio between bacterial and eukaryotic proteins (Bact:Euk) was about 1 (Fig. 4A). In the 0.2–0.8 μm size-fraction, however, the Bac:Euk ratio of proteins was 3, and in the <0.2 μm fraction ~5. The increase in the Bac:Euk ratio of proteins towards the smaller size-fractions, particularly in the bathypelagic (Wilcoxon test *p* < 0.05, Supplementary Data 5), indicates a shift in the source of organic matter, where eukaryotic and bacterial proteins contribute equally to the particle fraction but bacterial proteins dominate the dissolved protein pool.

By grouping eukaryotic proteins into taxonomic categories (zooplankton, algae, and fungi, Table S4), changes in eukaryotic protein profiles were observed throughout the water column (Figs. 4B, S6, Supplementary Data 6). Zooplankton-derived proteins exhibited a weak depth-related trend in the >0.8 μm size-fraction (Wilcoxon test, Epi- vs. Mesopelagic, *p* = 0.095; Epi- vs. Bathypelagic, *p* = 0.111, Fig. 4B Supplementary Data 5). In the meso- and bathypelagic, however, the relative abundance of zooplankton proteins (ca. 30%) was three times higher than algal proteins (5–10%) in both, the >0.8 μm and 0.2–0.8 μm size-fraction (Wilcoxon test, *p* < 0.05, Fig. 4B). This difference is in sharp contrast to the epipelagic, where the relative abundances of algal and zooplankton proteins were similar in the >0.8 μm size-fraction (Wilcoxon test, *p* > 0.05, Fig. 4B). Especially, in the >0.8 μm size-fraction, the relative abundance of algal proteins significantly decreased from the epipelagic (median = 14.0%, IQR = 12.7–18.5%) to the bathypelagic layer (median = 6.3%, IQR = 4.0–7.0%) (Wilcoxon test, *p* < 0.05).

Proteins are an essential component of total biomass and the changes in protein source reflect the variation in organic matter supply^37^. The attenuation of algal proteins with water column depth is consistent with the fact that sinking phytoplankton are insufficient to sustain the deep-sea microbiome^4,9^. It has been suggested that zooplankton-derived POM (fecal pellet, carcasses) and DOM^7,43,44^ become the primary carbon source in the deep sea, supporting microbial activity in the bathypelagic ocean^4^. This also implies that sequestration mechanisms like the gravity pump (fast-sinking zooplankton fecal pellet and carcasses packed into sinking particles)^8,43^ and the zooplankton migration pump (living zooplankton, 150–500 m)^3,45^ substantially contribute to the carbon flux into the meso- and bathypelagic ocean^2,3,7^. These different carbon pumps directly influence deep-sea POM’s quantity and composition such as marine snow^37^.

### Gammaproteobacteria are an essential source of bacterial protein

Alpha- and Gammaproteobacteria were the two major heterotrophic bacterial groups with gammaproteobacterial proteins dominating all three size-fractions, especially the <0.2 μm fraction, which is in contrast to the 16S rRNA profile (Figs. 4C, S7A, Supplementary Data 7). The ratio between Gammaproteobacteria and Alphaproteobacteria in the metaproteome in all size-fractions was significantly higher than the 16S rRNA-based ratio (Wilcoxon test, *p* < 0.05, Fig. S7B), suggesting that Gammaproteobacteria substantially contribute to protein production despite their relatively low abundance. Taxonomic analysis showed that while Alteromonadales and Oceanospirillales were the major contributors to gammaproteobacterial proteins in all fractions, the dominating groups in Alphaproteobacteria varied between fractions (Fig. S8A, B). This variability led to differences in the ratio of Gamma-/Alpha-proteobacterial proteins between fractions (Fig. S7B). For example, the cell abundance (16S rRNA gene) of the most abundant Alphaproteobacterium in the 0.2–0.8 μm fraction, Pelagibacterales (SAR11), was almost 30 times higher than Alteromonadales in the epipelagic (Fig. S9A). However, their protein abundance was lower than that of Alteromonadales throughout the water column (Fig. S9B). Cell-size measurements showed that deep-sea *Alteromonas* spp. had a larger (1.2 times) biovolume than Pelagibacterales (SAR11) (Wilcoxon test, *p* < 0.05, Fig. S9C, D). Thus, the smaller biovolume of SAR11 than Alteromonadales might have resulted in a low protein yield. This is consistent with the microfluidic mass sensor-based analysis that the dry mass of SAR11 is five- to twelve-fold lower than *Prochlorococcus* and almost two orders of magnitude lower than *Vibrio* (*V. splendidus* strain 13B01)^46^. As proteins account for about 50–60% of bacterial dry weight^47^, protein abundance can be used as a proxy for microbial biomass and add additional value in parameterizing ecological and biogeochemical models^48^.

Remarkably, gammaproteobacterial proteins dominated (ca. 80%) the <0.2 μm size-fraction (Figs. 4B, S8C, D). While proteins collected in the >0.8 μm and 0.2–0.8 μm size-fraction originated mainly from intact cells, proteins collected in the <0.2 μm fraction consisted of cell-free extracellular enzymes and proteins released from microorganisms. In our dataset, cell-free extracellular enzymes and protein debris in the <0.2 µm fraction were primarily of gammaproteobacterial origin (Fig. S8C, D). Signal peptides indicate protein/enzyme secretion into the environment^49^ and the yields of extracellular enzymes indicate their major role in POM solubilization and assimilation^16,50^. Ten to 15% of the proteins in the <0.2 μm fraction was associated with signal peptides and hence, were actively secreted as cell-free extracellular enzymes (Fig. S10A). In contrast, cell-associated extracellular enzymes detected in the 0.2–0.8 μm and >0.8 μm size-fraction varied in their relative abundance (Fig. S10B, C), but the functional composition of the cell-free and cell-associated extracellular enzymes was similar (Fig. S10D–F). In the extracellular enzyme pool, hydrolytic extracellular enzymes only accounted for <20% of the extracellular enzyme pool (Fig. S10D–F). Oxidoreductases involved in the oxidative degradation of algal polysaccharides^51^, in producing reactive oxygen species and mediating metal bioavailability^52^ contributed >40% to the extracellular enzyme pool (Fig. S10D–F). Oxidoreductases dominated both the extracellular and cytoplasmic enzyme pool (Fig. S10G–I) but their composition differed (Fig. S11), with cell-free oxidoreductases in the <0.2 μm fraction mainly acting on the CH-OH group (EC 1.1). Such functional difference suggests distinct enzymatic activities in extracellular substrate processing and cellular metabolism. Notably, the cell-associated extracellular enzymes might contribute to the cell-free extracellular enzyme pool when the cell is lysed (either due to viral lysis or cell decay), leading to overestimating the cell-free extracellular enzyme pool. However, we found significant differences between cell-associated and cell-free extracellular enzymes, which makes it unlikely that previously cell-associated extracellular enzymes contributed substantially to the dissolved enzyme pool. For example, although oxidoreductases were the most abundant extracellular enzymes in all fractions (Fig. S10D–F), in the 0.2–0.8 µm fraction, the enzyme class EC1.17 was relatively abundant but hardly contributed to <0.2 m or >0.8 µm fraction (Fig. S11A). Similarly, the enzyme class EC1.8 was found in both, the 0.2–0.8 µm and >0.8 µm fractions but was barely detected in the <0.2 µm fraction (Fig. S11A). Such changes suggest marginal interference of cell-associated extracellular enzymes to the cell-free extracellular enzyme pool and show metabolic adaptations of microbes present in the different size fractions (0.2–0.8 µm vs. >0.8 µm).

As zooplankton proteins are a major particulate protein source in the deep ocean (Fig. 4B), the dominance of extracellular enzyme production suggests that Gammaproteobacteria utilize zooplankton-derived POM, which might support their cellular metabolism either for growth, respiration and/or mitigate oxidative stress caused by high hydrostatic pressures^17^. Leucine incorporation rates, used as a proxy for heterotrophic microbial activity, revealed that the metabolic activity of *Alteromonas* is, although reduced by the high hydrostatic pressure in the deep ocean, still comparable to pressure tolerant groups like SAR202^17^. Leucine incorporation rates of *Alteromonas* spp. contributed 25–50% of the mean leucine incorporation rate in the deep ocean (Fig. S9E, Supplementary Data 10), but the cell abundance of *Alteromonas* spp. was relatively low (~10^3^cells ml^−1^)^18,53^, which suggests disproportionally high cell loss. Consistently, we found that proteins released from cell decay (proteins without signal peptide) accounted for 80% of the dissolved protein pool in the <0.2 μm size-fraction (Fig. S10A), where Alteromonadales contributed 40–50% of dissolved gammaproteobacterial protein (Figs. 4C, S8D). This protein percentage indicates that lysed Alteromonadales cells might be a major source for the dissolved protein pool.

Viral lysis and zooplankton grazing are the major causes of cell death in microbes^8,19,27^. High viral lysis rates were observed in marine detrital particles as a significant fraction of deep-sea heterotrophic microbes preferentially associated with particles^16,54^. Genetic analysis suggests that particle attached-microbes have a higher growth efficiency than free-living microbes in the deep sea^55,56^. Thus, the lytic infection of active Gammaproteobacteria would efficiently convert cellular organic matter into DOM due to viral lysis^19,57^. In our dataset, viral proteins constituted 1–5% of the total proteome (Fig. 4A), with Myo-, Podo-, and Siphoviridae comprising most of the viruses (Fig. S12A). About 3–18% of viral proteins expressed by viruses could be linked to putative hosts (Supplementary Data 8). Linking viral proteins to the putative host (see Methods, Figs. 4D, S12B, C) revealed that the relative abundance of viruses infecting Gammaproteobacteria was highest in all fractions (viruses reproducing in the cell, Fig. 4D, Wilcoxon test, *p* < 0.05, Supplementary Data 9). Viral proteins detected in the >0.2 μm (>0.8 μm and 0.2–0.8 μm, Fig. 4C) fraction might be viruses actively infecting host cells^42,58^. Recent metagenomic examination of cell-associated viral communities in the deep ocean indicate that Alpha- and Gammaproteobacteria are the major hosts of deep-sea viruses^59^. A remarkable niche separation has also been reported in host preference of deep-sea viruses where Gammproteobacteria are primarily hosts on particles^59^. However, no metagenomic data are available from the <0.2 µm fraction in the deep ocean, which might lead to underestimating deep-sea viruses. Viruses rely entirely on their host cells’ translation machinery to produce proteins like capsid, essential for viral replication. Previous results showed that the transcripts of host ribosomal protein become upregulated within the first hour of viral infection and are probably released together with viral progenies during the lytic process^58^. In our dataset, the high relative abundance of ribosomal proteins in the <0.2 µm fraction (Fig. 3) provides a strong indication of viral intervention on the host machinery for viral propagation^60,61^, where viruses use bacterial ribosomal protein to synthesize viral proteins and the ribosomal protein is released into ambient water after cell lysis. The high relative abundance of viruses infecting Gammaproteobacteria in the >0.8 µm and 0.2–0.8 µm fractions suggests an active generation of progeny viruses in gammaproteobacterial cells, eventually resulting in cell lysis and release of gammaproteobacterial cellular proteins into the <0.2 μm size-fraction (Figs. 4C, S8D). Potentially, Gammaproteobacteria might be more “fragile” than other bacteria, and cells might break during filtration, which would have biased our conclusion. However, in our filtration process, we applied 1.5–2.0 bar air pressure, much lower than pressures necessary for cell disruption (French press, 1300–2700 bars). Hence, cell rupture of specifically, Gammaproteobacteria is highly unlikely. Also, Gammaproteobacteria cannot easily pass through the 0.2 μm filter pores as SAR11 is much smaller than Alteromonadales, which were well retained by our filtration setup. Contamination due to growth in the filtrate is also unlikely because the growth rate of Gammaproteobacteria in unamended deep-sea waters is very slow^6^, and we used SDS for growth inhibition in the <0.2 µm fraction.

To further examine the possible contribution of Gammaproteobacteria to the DOM pool, we analyzed the metagenomic profile from the <0.2 µm fraction collected by the *Tara* Ocean expedition^27^. We also found a high relative abundance of gammaproteobacterial DNA in the metagenomes of the <0.2 μm fraction in the epipelagic realm with a taxonomic profile similar to our metaproteome (Fig. S13A). Classification at the order level showed that Sphingomonadales and Rhodobacterales from Alphaproteobacteria (Fig. S13B), Alteromonadales and Oceanospirillales from Gammaproteobacteria (Fig. S13C) were the major groups in the metagenomes of the <0.2 μm fraction, which is consistent with our metaproteome data (Fig. S8). This further indicates Gammaproteobacteria’s high contribution to the DOM pool, which starkly contrasts their 16S rRNA based relative abundance (Fig. S7A). Thus, the cellular components released to the DOM pool, together with the high yields of extracellular enzymes, indicate a thus far overlooked role of Gammaproteobacteria in the deep ocean’s carbon cycle (Fig. 4A, B). However, this conclusion has to be taken with caution as no direct lytic activity was detected on Gammaproteobacteria.

A close virus-host interaction was also found for Cyanobacteria in the epipelagic layer (Fig. 4D). Cyanobacteria were most abundant in the >0.8 μm size-fraction (ca. 30%) in the epipelagic and decreased in relative abundance with depth (ca. 2% in the bathypelagic, Fig. 4C, Supplementary Data 6). Along with the cyanobacterial hosts, cyanophages were abundant in the epipelagic waters in the >0.8 µm and <0.2 µm fraction (Fig. 4D), indicating intensive viral infection of cyanobacteria. The relative abundance of the viral photosystem-II (psbA) was similar to the relative abundance of cyanobacterial psbA, especially in the >0.8 μm size-fraction (Figs. 4B, C, S12D) reflecting close virus-host interactions^58^. These results are consistent with those from metatranscriptomic analyses, where 50% of psbA transcripts originated from cyanophages, confirming the major role of viruses in regulating photosynthetic processes in the sunlit surface ocean^62^. Stable isotope labeling proteomics^63,64^ together with lysis rate measurements^54^ will provide an in-depth view on virus mediated carbon flux in the global ocean.

Bacteroidetes derived proteins were also abundant in the epipelagic but decreased in abundance with depth (Wilcoxon test, *p* < 0.05) in both the >0.8 μm and 0.2–0.8 μm size-fraction (Fig. 4C), consistent with the results of 16S rRNA analysis (Fig. S7A). Recently, it has been shown that high hydrostatic pressure substantially inhibits the metabolic activity of Bacteroidetes and *Alteromonas* in the deep sea^17^; however, compared to *Alteromonas*, Bacteroidetes suffers higher oxidative stress under high pressure conditions^17^. Also, Bacteroidetes need excessive trimethylamine to maintain their morphology in the deep sea^65^. The different adaptive mechanisms of *Altermonas* and Bacteroidetes might shape the distinct depth profile throughout the water column.

### Urea fuels nitrification-mediated dark inorganic carbon fixation

**Fig. 5:**
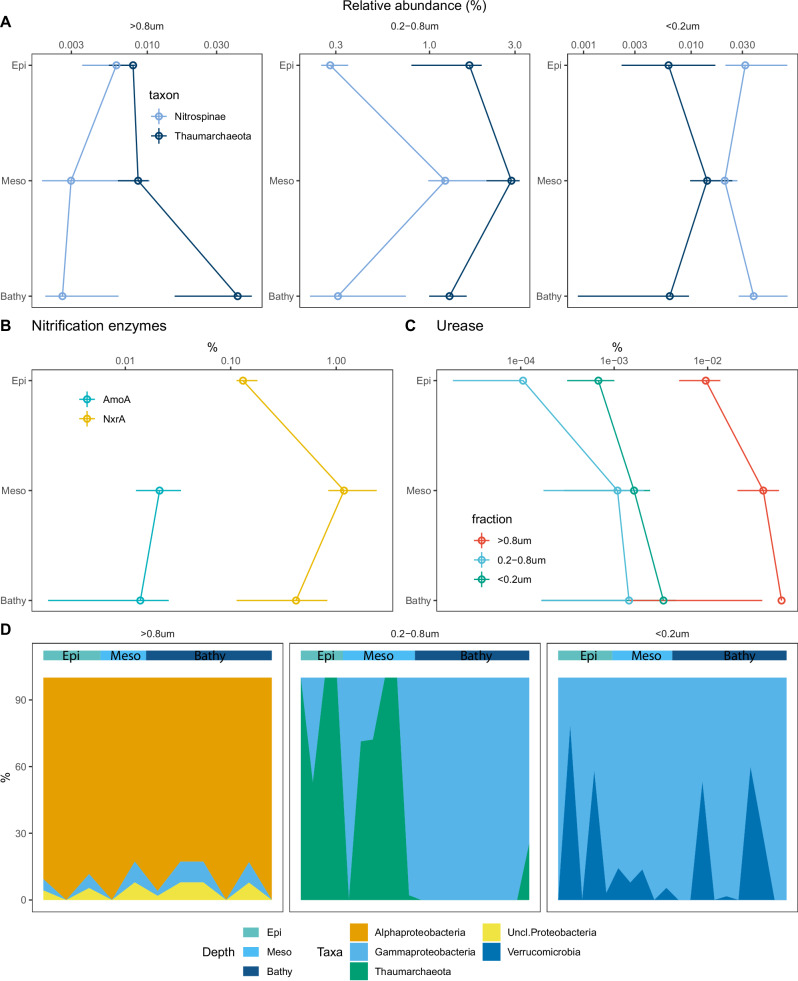
Nitrification process revealed in the metaproteome. **A** Relative abundance of proteins of nitrifiers in each size-fraction. **B** Relative abundance of key enzymes involved in nitrification in the 0.2–0.8 μm size-fraction. **C** Relative abundance of urease in each size-fraction. **D** Taxonomic breakdown of urease in each size-fraction. The *x*-axis represents metaproteomic samples from different stations, and the depth layer is indicated in the horizontal color bar on top of the panels. The points and ranges show the medians, the 25th and 75th IQR. Epi, samples collected from epipelagic (<200 m, *n* = 15); Meso, samples collected from mesopelagic (200–1000 m, *n* = 16); Bathy, samples collected from bathypelagic (>1000 m, *n* = 30); >0.8 μm, samples collected from the >0.8 μm fraction (*n* = 19); 0.2–0.8 μm, samples collected from the 0.2–0.8 μm fraction (*n* = 22); <0.2 μm, samples collected from the <0.2 μm fraction (*n* = 20). Source data are provided as a Source Data file.

Thaumarchaea and Nitrospinae were the major chemolithoautotrophs in our metaproteome, with the highest relative abundances detected in the 0.2–0.8 μm size-fraction in the mesopelagic (Fig. 5A). The relative abundance of Nitrospinae in the metaproteome was higher than expected from the 16S rRNA analysis (Figs. 5A, S7C, D) because Nitrospinae cells are larger compared to other bacterial taxa^15^. In contrast, thaumarchaeal cells have a small biovolume, as revealed by microscopic analyses (only 60% of SAR11, Fig. S9C, D). Ammonia monooxygenase (AmoA) and nitrate oxidoreductase (NxrA) are the key enzymes used by Thaumarchaea and Nitrospinae, respectively, for energy harvesting to fuel dark DIC fixation. In our metaproteome, the relative abundance of NxrA was almost two orders of magnitude higher than that of AmoA (Fig. 5B), which compensates for the lower abundance of Nitrospinae compared to Thaumarchaea (Fig. S7C). Caution should be paid that tryptic digestion and ionization efficiency may differ between proteins/peptides from different taxonomic/functional groups. Still, the high expression of NxrA in our analysis supports previous reports that NxrA is one of the most abundant oxidoreductases in the mesopelagic^20,66^. Nitrospinae also exhibits one order of magnitude higher DIC fixation rates^15^ but lower (three- to four-fold) energy conversion efficiency^67^ than Thaumarchaea. Thus, a high cell-specific oxidation rate is suggested for Nitrospinae and the high expression level of NxrA (Fig. 5B) found in our dataset suggests homeostasis of the nitrogen flux between Nitrospinae and Thaumarchaea^67^. However, the difference in the mortality rate between Thaumarchaea and Nitrospinae was also suggested as an alternative explanation for differences in cell abundance and energy thermodynamics in oxygen depleted areas^68^.

The first nitrification step is ammonia oxidation, providing energy for DIC fixation. Previous reports suggest that, due to insufficient ammonia supply, urea might serve as an alternative ammonium source for Thaumarchaea as they can use urease for urea cleavage^14,20,53^. In the 0.2–0.8 μm size-fraction almost 90% of the urease present in the mesopelagic (Fig. 5C, D) was expressed by Thaumarchaea on the metaproteome level, which reached their highest relative abundance in the mesopelagic layers (Figs. 4A, 5A, S7C). This high contribution of thaumarchaeal urease coincided with the high contribution of zooplankton-derived proteins to the total protein pool in the mesopelagic realm (Fig. 4B). In the open ocean, zooplankton can excrete large amounts of urea and its concentration is significantly correlated with zooplankton biomass^10,69^. Nitrifiers like Thuamarchaea can directly use the urea excreted by copepods^12^. Thus, the activity of zooplankton in the mesopelagic ocean might provide POM for heterotrophs and indirectly support dark DIC fixation of Thaumarchaea via the release of urea. Although the highest relative abundance of thaumarchaeal urease was found in the mesopelagic in the 0.2–0.8 µm fraction, we also detected thaumarchaeal urease in the deep-sea (Fig. 5D). Genes encoding thaumarchaeal urease were found in deep-sea metagenomes^70^ and Thaumarchaea contributes 10–20% to the total prokaryotic production in Atlantic deep waters^53^. The detection of thaumarchaeal urease in the bathypelagic metaproteome further supports the hypothesis that deep-sea Thaumarchaea also use urea as an ammonia source for DIC fixation^53,70^. Nitrospinae was also reported to use urease to cleave urea^15,68^, but Nitropinae related urease was not found in our metaproteome. We constructed a phylogenetic tree (Fig. S14) of UreC protein sequences identified in our metaproteomics together with reference UreC sequences of Gammaproteobacteria (Alteromonadales, Oceanospirilalles), Alphaproteobacteria (SAR11, Rhodobacterales, Rhodospirilalles), AOA (*Nitrosopumilus*), Cyanobacteria (*Synechococcus*) and Nitrospinae^68,71^, the result showed that although the phylogenetic placement of Nitrospinae-UreC sequences was close to the gammaproteobacterial UreC, but none of the identified UreC from our analysis was placed into the Nitrospinae-UreC cluster. We further added the Nitrospinae-UreC sequences to our database to check whether any peptide overlooked from our analysis due to the low number of Nitrospinae-UreC sequences in the database, but still, no Nitrospinae-UreC related peptide detected. Such results are similar to other metaproteomic studies^15,20^, indicating a low abundance of Nitropinae-related urease in the open ocean. Future metaproteomic sampling in marine oxygen minimum zones might provide stronger signal for Nitrospinae urease as the gene and the enzymatic activity are primarily reported in the oxygen depleted seawaters^68,71^.

In addition, niche partitioning was found in heterotrophic bacterial urea utilization, where urease in the >0.8 μm size-fraction was expressed by Alphaproteobacteria while gammaproteobacterial urease dominated the <0.2 μm size-fraction (Fig. 5D). As bacterial urease is located in the cytoplasmic space, the dominance of gammaproteobacterial urease in the <0.2 µm fraction further suggests that Gammaproteobacteria are actively utilizing urea when the cell is intact and release urease into ambient seawater after cell lysis. This supports our conclusion that active Gammaproteobacteria may be exposed to high viral lysis rates.

We also detected denitrification related enzymes such as dissimilatory nitrate reductase (NapA/NarG) in the metaproteome (Fig. S15C, D). In general, the denitrification enzymes exhibited the highest relative abundance (0.3–0.5% of the total proteome) in the >0.8 µm fraction (Fig. S15C). This supports the hypothesis that detrital particles provide a niche for anaerobic microbial metabolism in the dark ocean^72^. Enzymes involved in aerobic respiration (CoxA/CyoA/CcoN/CydA, cytochrome oxidase) were drastically reduced in the >0.8 μm size-fraction in the mesopelagic (Fig. S15A, B) compared to the epipelagic waters. However, only NapA, NarG, and NirK were found in the >0.8 µm fraction. In contrast, Nitrous Oxide Reductase (NosZ) and hydrazine synthase (HzsA), two typical enzymes involved in anaerobic denitrification, were found in the 0.2–0.8 µm and <0.2 µm fraction. The relative abundance of denitrification enzymes in the 0.2–0.8 µm and <0.2 µm fraction was 0.05% and 0.02% of the total proteome, respectively (Fig. S15D), NosZ and HszA only contributed <0.5% to the total denitrification enzymes. Phylogenetic analysis further revealed that the NosZ protein sequence detected in our metaproteome showed high similarity (Fig. S15E) to known NosZ sequences. This suggests the capability of marine bacteria to convert N_2_O to N_2_, but the microenvironment might primarily determine the expression. Also, the NosZ in the dissolved fraction (<0.2 µm) might originate from cell lysis because NosZ is a cell-associated extracellular oxidoreductase, and it contributed marginally to the dissolved protein pool (1 × 10^−4^%). A similar analysis was also made for HszA (Fig. S15F). The HszA sequences in the metaproteome were similar to hydrazine synthase in *Ca. Scalindua rubra* [ODS33869.1].

Knowledge on the composition and functional variability of the ocean’s microbiome is crucial to understanding the biogeochemical processes in the different strata of the water column^1^. By characterizing and quantifying the protein abundance of the entire microbial consortia, our metaproteomic analyses provide semi-quantitative results on the taxonomic contribution to the marine protein pool. Our metaproteomic approach revealed that zooplankton detritus contributes about 30% to the eukaryotic protein pool in the meso and bathypelagic ocean (Fig. 4B), and likely serves as a major POM source (zooplankton migration pump, fecal pellet and carcasses packed into sinking particles) dominating over phytoplankton flux in the meso- and bathypelagic ocean.

Besides providing POM, urea released from zooplankton might be one of the major ammonia sources supporting nitrification in the mesopelagic zone, as indicated by the relatively high abundance of thaumarchaeal urease at depth (Fig. 5). The expression of thaumarchaeal urease in the 0.2–0.8 µm fraction in the mesopelagic provides support for the tentative link between nitrification and zooplankton activity^12,14^. Hence, dark DIC fixation in the mesopelagic is likely supported by the zooplankton release of urea, ultimately resulting in additional organic carbon becoming available to the microbial community in the dark ocean. We also found significant differences between the relative abundance of nitrification enzymes (AmoA vs. NxrA). These results provide insight into the DIC fixation in the mesopelagic mediated by marine nitrifiers.

Gammaproteobacteria primarily utilize POM by secreting extracellular enzymes (Figs. 4C, S8C). Gammaproteobacteria also maintain their metabolic activity under high pressure conditions (Fig. S9C). Despite their relatively low abundance (~10^3^ cells ml^−1^)^18,53^, Gammaproteobacteria contribute 10–30% to the bacterial protein pool in the 0.2–0.8 µm fraction (Fig. 4C). However, they also experience significant cell losses via grazing pressure and/or viral lysis (Fig. S8D). The detection of viral proteins in the size-fractions >0.8 µm and 0.2–0.8 µm together with the high relative abundance of ribosomal proteins in the <0.2 µm fraction suggests dynamic viral-host interactions between gammaproteobacterial hosts and their corresponding viruses (Figs. 3, 4C, D). The viral lysis of active Gammaproteobacteria appears to be critical in converting cellular organic matter into DOM. Eventually, it contributes to the bioavailable DOM pool in the dark ocean, a process known as the viral shunt^57,73^.

Our characterization of the bulk microbiota at the protein level provides holistic and direct information on trophic interactions mediating the carbon flux in the deep ocean. Our data suggest that zooplankton-derived POM and inorganic nutrients substantially support the carbon cycle in the meso- and bathypelagic oceans. We also provided evidence on how the metabolic activities of heterotrophic and autotrophic microbes, together with viral lysis convert POM and inorganic nutrients into labile DOM.

## Methods

### Sampling and filtration

About 100–400 L of seawater were sequentially filtered through 0.8 μm and 0.2 μm pore-size polycarbonate membranes (Isopore, 142 mm diameter, Millipore) at 22 stations in the Pacific, Atlantic and Southern Ocean (Fig. S1A, Supplementary Data 1). Two large volume filtration holders (Sartorius) in conjunction with diaphragm pumps operated at a positive pressure not exceeding 1.5–2.0 bars were used. The filtration process generally finished within 1.5–2 h. The filtrates were amended with SDS to a final concentration of 0.1% (*w/w*) to avoid protein aggregation and inhibit bacterial growth. The 0.2 µm filtrate was further concentrated with tangential flow filtration driven by peristaltic pumps and using low protein binding membranes of a molecular weight cutoff at 5000 Da. To reach a final volume of ~50 ml of viral and dissolved proteins, an 0.5 m^2^ ultrafiltration cassette (Pellicon 2 Ultracel membrane, Millipore) and a 200 cm^2^ polyethersulfone cartridge with a hold up volume <2 ml (Vivaflow 200 module, Sartorius) were combined. The filters and the concentrates were immediately frozen in liquid nitrogen and stored at −80 °C until extraction. Eukaryotic detritus like algal aggregates, zooplankton carcasses and fecal pellets, cyanobacteria, and particle-attached heterotrophic bacteria were mainly collected in the >0.8 μm fraction and free-living prokaryotes in the 0.2–0.8 μm fraction; viruses together with dissolved proteins/enzymes, either secreted or released, were obtained in the <0.2 μm fraction^27,30,74,75^.

### Metagenomics and metaproteomics extraction and sequencing

From the 0.2–0.8 μm filters, slices of about one-eighth to one-quarter were used for DNA extractions, corresponding to ~20 L of sample^76^. Filters were lysed with a buffer containing 0.75 M sucrose, 50 mM Tris-HCl (pH = 8), 40 mM EDTA (pH = 8), lysosome (1 µg ml^−1^), ProteinaseK (2.5 mg ml^−1^) and 5% SDS. The supernatant was cleaned with phenol:chloroform:IAA (25:24:1) and precipitated with 35% isopropanol. The total DNA was sequenced on an Illumina NextSeq 500 platform. Protein extraction from the remaining filter was performed in the lab using lysis buffer containing 7 M urea, 2 M thiourea, 1% DTT, 2% CHAPS, and protease inhibitor cocktail. The mixture was homogenized with bead-beating, sonicated at high power with pulses of 10 s over 10 min. The supernatant from the slurry and the concentrates were separately concentrated to 250 μL with a 3000 Da Amicon Ultra-15 Centrifugal Filter Unit (Millipore). The protein fraction was precipitated with cold ethanol overnight at −20 °C, and resuspended with 7 M urea and 2 M thiourea. The protein pellet of each sample was digested using the filter-aided in-solution trypsin digestion method (1:100, *w*/*w*)^77^. The tryptic peptide pellets were dissolved in 4% (v/v) acetonitrile, 0.1% (v/v) formic acid. After desalting (C18) the peptides were sequenced on a Q-Exactive™ Hybrid Quadrupole-Orbitrap™ Mass Spectrometer (ThermoFisher Scientific). At least 2 replicates per sample were loaded on C18 reverse-phase columns (EASY-Spray 500 mm, 2 µm particle size, ThermoFisher Scientific). Separation was achieved with a 90 min gradient from 98% solution A (0.1% formic acid in high purity water) and 2% solution B (90% ACN and 0.1% formic acid) at 0 min to 40% solution B (90% ACN and 0.1% formic acid) at 90 min with a flow rate of 300 nL min^−1^. Nano-electrospray ionization MS/MS measurements were performed with the following settings: Full scan range 350–1800 m/z resolution 120,000 max. 20 MS2 scans (activation type CID), repeat count 1, repeat duration 30 s, exclusion list size 500, exclusion duration 30 s, charge state screening enabled with the rejection of unassigned and +1 charge states, minimum signal threshold 500. The mass spectrometry proteomics data were deposited to the ProteomeXchange Consortium via the PRIDE^78^ partner repository with the dataset identifier PXD034421.

### Acquisition of gene catalogs for the marine planktonic community

Marine eukaryotic and viral sequences were downloaded from previous publications^27,30^. A prokaryotic gene catalog was construct from metagenomics reads downloaded from the National Center for Biotechnology Information (NCBI) website, together with an in-house database of sequencing results (Supplementary Data 2). Reads from the metagenomic dataset were assembled individually using Megahit v1.1.2 with default settings^79^. Subsequently, putative genes were predicted on contigs longer than 200 bp using Prodigal version 2.6.3 under metagenome mode (-p meta)^80^ and further clustered at 90% similarity (-c 0.9 -G 0 -aS 0.9) using CD-HIT v4.6.8^81^ to construct the prokaryotic gene database for downstream metaproteomic analysis. To retrieve the relative abundance of prokaryotic taxa in the metagenome, miTAG analysis^82^ was performed by extracting 16S rRNA genes using SortMerRNA^83^ for downstream analysis with LotuS^84^. Analysis of gene-based operational taxonomic units (mOTUs)^85^ was also performed for both, the metagenomics and metatranscriptomic datasets to cover the microbial abundance and activity, respectively.

### Proteomic annotation and analysis

The construction of a robust database is key to interpret metaproteomic samples, thus we used an optimized database construction strategy^28^. We combined the predicted genes from our in-situ metagenomics assembly with publicly available gene catalogs from metagenomics/metatranscriptomic assemblies of the global ocean Tara, Malaspina, and Bio-Geotraces expeditions^21,24,25,30^ to obtain a deep coverage of microorganisms (including micro-eukaryotes and viruses) throughout the entire water column. All these sequences were concatenated and de-replicated to construct a non-redundant database to avoid biases by introducing any over-represented sequences. All mass spectrometry files from different size fractions were searched against the same database. Due to the large size of the gene catalogs a two-step database searching method was used^29^. Briefly, in the first search, the MS/MS spectra of proteomic samples were pooled and searched using the SEQUEST-HT^86^ engines against proteins in the databases of eukaryotes, prokaryotes, and viruses with a loose false discovery rate (FDR) of 10%. Sequences identified in this step were exported to a refined database for the second search where the proteomic samples were analyzed separately. In this step the FDR was set to 1% for protein selection and the scoring function Xcorr threshold was set to 1 per charge (2 for +2 ions, 3 for +3 ions, etc.). The variable modifications were set to acetylation of the N-terminus and methionine oxidation, with a mass tolerance of 10 ppm for the parent ion and 0.8 Da for the fragment ion. We allowed 2 missed and non-specific cleavages and only dynamic modifications were used. Percolator in Proteome Discoverer 2.1 (ThermoFisher Scientific) was used for validation. A minimum of two peptides and one unique peptide were required for protein identification, no protein grouping was used in this analysis. Functional annotation of identified protein sequences was performed by searching against EggNOG^87^, KEGG^36^ and Pfam^88^, using emapper^89^. Taxonomic affiliation of sequences was determined using the lowest common ancestor algorithm (LCA, diamond blastp --top 10 –sallseqid -outfmt 102) adapted from DIAMOND v2.0.9^90^ blast by searching against the non-redundant (NR) database (downloaded from NCBI in March 2022). The top 10% hits with an *e*-value < 1 × 10^−5^ were used for taxon determination (--top 10). Metabolic annotation for proteins/enzymes involved in photosynthesis, nitrification, denitrification and respiration was done using DIAMOND v2.0.9^90^ (--max-target-seqs 1, --max-hsps 1) searching against metabolic marker protein databases (https://bridges.monash.edu/collections/_/5230745)^91^. Since these are highly conserved sequences, they require a high level of discrimination to differentiate them (i.e., NxrA and NarG share the same KO). SignalP v5.0^49^ was used to detect the presence of signal peptides for extracellular enzymes of bacterial origin. The gram-positive mode was used for sequences affiliated to Actinobacteria and Firmicutes. Sequences with hits on COG0804 |COG0831|COG0832|COG0829| COG0378 in the EGGNOG database and/or K01427 |K01428|K01429|K01430| K14048 in the KEGG annotation were kept as urease and the taxonomic affiliation of urease was determined via LCA analysis as described above. Sequence alignment of UreC sequences was conducted using MAFFT (online server, version 7, with default setting)^92^. Phylogenetic tree of ureC sequences was constructed using FastTree (2.1)^93^ and visualized using Interactive Tree of Life (iTOL, v6)^94^. All ureC sequences for the phylogenetic analysis are available via FigShare (10.6084/m9.figshare.24570104). Virus-host prediction was achieved by blasting viral proteins against predicted viral genes derived from previous reports on marine viral communities collected from the entire water column^27,59^. We assumed that viral proteins with similarity >90% share the same host. Protein quantification was conducted with the normalized area abundance factor (NAAF), a chromatographic label-free method based on peak area^95^. The NAAF is calculated as:

1 $${NAAF}=\frac{{x}_{i}}{{L}_{i}}/\sum \frac{{x}_{i}}{{L}_{i}}$$

Where *x*_*i*_ represents the peak area of a peptide and *L*_*i*_ represents the length of the peptide. Only the peak area of unique peptides (a peptide not shared with other proteins or protein groups) and Razor peptide (a peptide shared by multiple different proteins will be assigned to the proteins with the highest number of unique peptides but with the shortest protein length) was employed for the quantitation.

### Measurements of prokaryotic cell size

Cell sizes of prokaryotes used here were analyzed in the study of Amano et al.^17^ by measuring the area of 4′,6-diamidino-2-phenylindole (DAPI)-stained cells that in addition were labeled with catalyzed reporter deposition fluorescence in situ hybridization (CARD-FISH) using group specific oligonucleotide probes. Briefly, the CARD-FISH samples were collected from ~450–4000 m at 7 stations in the Atlantic and Southern Ocean where 4 stations overlap with the sampling stations of the metaproteomic analysis (Supplementary Data 1). Target organisms filtered onto 0.2 µm-pore size filters were visualized with the 5’-horseradish-peroxidase-labeled oligonucleotide probes: Alt1413 probe for *Alteromonas/Colwellia*^96^, a mix of SAR11-152R, SAR11-441R, SAR11-542R and SAR11-732R probes for the SAR11 clade^97^ and a mix of Cren537 and GI-554 probes for Thaumarchaea^98,99^. Original images were taken on an epifluorescence microscope (Axio Imager M2, Carl Zeiss). Image analysis was conducted with the ACMEtool3 (Zeder, M. 2005-2021, Software for Biology, http://www.technobiology.ch) by segmenting the DAPI signals from the 8-bit grayscale images and sorting CARD-FISH positive signals. After manually checking the detection of the cells and their morphology, the cell volume (*V*) was calculated via the area size and perimeter of each DAPI signal by assuming the rod-model:

2 $$V=\pi {r}^{2}\cdot \left(l-2r\right)+\frac{4}{3\pi {r}^{3}}$$

where *r* is radius of a cell and *l* is the length of a cell. Although size estimates using DAPI can be smaller than the actual cell size as discussed previously^100,101^, we found that DAPI-stained cell volumes correspond to the amount of DNA in a cell and to the cell biomass^102^.

#### Determination of cell-specific leucine incorporation rates

Cell-specific leucine incorporation rates were measured in a previous study^17^. Briefly, the size of the silver grain halo around each DAPI-positive cell was measured using Axio Vision SE64 Re4.9 (Carl Zeiss) following microautoradiography performed on CARD-FISH processed samples (MICRO-CARD-FISH). The halo areas were converted to leucine incorporation rates in mol day^−1^ with an equation obtained from correlating the total area of the halos with the bulk leucine incorporation rates^103^.

#### Statistical analysis and visualization

We used a machine-learning random forest^41^ classification to predict feature KOs between different size-fractions using *randomforest*^104^ package in R. Random forest classification was carried out with a relative abundance table of KOs from 61 samples. Only KOs with a relative abundance >=1% in at least one sample was kept as prediction features (231 out of 3817 KOs). The relative abundance table of KOs was randomly spilt into “training data” (containing 70% of the sample, 41 out of 61) and “testing data” (containing 30% of the sample, 20 out of 61). A random forest model was built using “training data” by classifying the relative abundance of KOs against size-fractions. KOs ranked by random forests according to feature importance were determined over 1000 iterations (ntree = 1000). The number of decision trees (ntree) was determined by Out-of-Bag (OOB) error for different values (ntree = c[100, 200, 500, 1000,1500]) of ntree. A minimal OOB error was found when ntree = 1000. The number of marker KOs were identified using a 10-fold cross-validation with the *rfcv()* function. The cross-validation error became stabilized when using at least the top 14 most important KOs (Fig. S5). The random forest model was further applied to the “test data” to examine prediction accuracy with the *predict()* and *confusionMatrix()* function. The accuracy, sensitivity, and specificity of the model can be found in the Supplementary Data 11. The classification rule for each decision tree is provided in Supplementary Data 12. Other statistics and visualization were also performed using packages in R. Specifically, *Vegan*^105^*, ggplot2*^106^*, circlize*^107^*, pheatmap*^108^ were used for ordination, diversity calculation, and visualization, respectively.

### Reporting summary

Further information on research design is available in the Nature Portfolio Reporting Summary linked to this article.

## Supplementary information

Supplementary Information

Description of Additional Supplementary Files

Supplementary Data 1–12

Reporting Summary

## Source data

Source Data

## Data Availability

Mass spectral data are available via ProteomeXchange with identifier PXD034421. Metagenomic reads have been deposited to the National Center for Biotechnology (NCBI) under Bioproject number PRJNA503889. The protein sequences used as metaproteomic database and metaproteomic results are available on FigureShare (10.6084/m9.figshare.24570104.v1). Source data are also provided with this paper. Source data are provided with this paper.

## Code availability

R scripts for Permanova analysis, random forest analysis, and different abundance analysis are available on github (https://github.com/zhaoz59/marine_metaP).

## References

[1] Azam, F. & Malfatti, F. Microbial structuring of marine ecosystems. *Nat. Rev. Microbiol* **5**, 782–791 (2007). 17853906 10.1038/nrmicro1747

[2] Ducklow, H. W., Steinberg, D. K. & Buesseler, K. O. Upper ocean carbon export and the biological pump. *Oceanography* **14**, 50–58 (2001).

[3] Boyd, P. W., Claustre, H., Levy, M., Siegel, D. A. & Weber, T. Multi-faceted particle pumps drive carbon sequestration in the ocean. *Nature* **568**, 327–335 (2019). 30996317 10.1038/s41586-019-1098-2

[4] Herndl, G. J. & Reinthaler, T. Microbial control of the dark end of the biological pump. *Nat. Geosci.* **6**, 718–724 (2013). 24707320 10.1038/ngeo1921 PMC3972885

[5] Jiao, N. et al. Microbial production of recalcitrant dissolved organic matter: long-term carbon storage in the global ocean. *Nat. Rev. Microbiol.* **8**, 593–599 (2010). 20601964 10.1038/nrmicro2386

[6] Arrieta, J. M. et al. Ocean chemistry. Dilution limits dissolved organic carbon utilization in the deep ocean. *Science* **348**, 331–333 (2015). 25883355 10.1126/science.1258955

[7] Moran, M. A. et al. The Ocean’s labile DOC supply chain. *Limnol. Oceanogr.* **67**, 1007–1021 (2022).

[8] Steinberg, D. K. & Landry, M. R. Zooplankton and the ocean carbon cycle. *Ann. Rev. Mar. Sci.* **9**, 413–444 (2017). 27814033 10.1146/annurev-marine-010814-015924

[9] Aristegui, J., Gasol, J. M., Duarte, C. M. & Herndl, G. J. Microbial oceanography of the dark ocean’s pelagic realm. *Limnol. Oceanogr.* **54**, 1501–1529 (2009).

[10] Eppley, R. W., Renger, E. H., Venrick, E. L. & Mullin, M. M. A study of plankton dynamics and nutrient cycling in the central gyre of the north Pacific Ocean 1. *Limnol. Oceanogr.* **18**, 534–551 (1973).

[11] Painter, S. C., Sanders, R., Waldron, H. N., Lucas, M. I. & Torres-Valdes, S. Urea distribution and uptake in the Atlantic Ocean between 50°N and 50°S. *Mar. Ecol. Prog. Ser.* **368**, 53–63 (2008).

[12] Valdés, V., Fernandez, C., Molina, V. & Escribano, R. Nitrogen excretion by copepods and its effect on ammonia-oxidizing communities from a coastal upwelling zone. *Limnol. Oceanogr.* **63**, 278–294 (2018).

[13] Wuchter, C. et al. Archaeal nitrification in the ocean. *Proc. Natl Acad. Sci. USA* **103**, 12317–12322 (2006). 16894176 10.1073/pnas.0600756103 PMC1533803

[14] Alonso-Saez, L. et al. Role for urea in nitrification by polar marine Archaea. *Proc. Natl Acad. Sci. USA* **109**, 17989–17994 (2012). 23027926 10.1073/pnas.1201914109 PMC3497816

[15] Pachiadaki, M. G. et al. Major role of nitrite-oxidizing bacteria in dark ocean carbon fixation. *Science* **358**, 1046–1051 (2017). 29170234 10.1126/science.aan8260

[16] Zhao, Z., Baltar, F. & Herndl, G. J. Linking extracellular enzymes to phylogeny indicates a predominantly particle-associated lifestyle of deep-sea prokaryotes. *Sci. Adv.* **6**, eaaz4354 (2020). 32494615 10.1126/sciadv.aaz4354 PMC7159927

[17] Amano, C. et al. Limited carbon cycling due to high-pressure effects on the deep-sea microbiome. *Nat. Geosci.* **15**, 1041–1047 (2022). 36504693 10.1038/s41561-022-01081-3 PMC9726642

[18] Agogue, H., Lamy, D., Neal, P. R., Sogin, M. L. & Herndl, G. J. Water mass-specificity of bacterial communities in the North Atlantic revealed by massively parallel sequencing. *Mol. Ecol.* **20**, 258–274 (2011). 21143328 10.1111/j.1365-294X.2010.04932.x PMC3057482

[19] Luo, E., Leu, A. O., Eppley, J. M., Karl, D. M. & DeLong, E. F. Diversity and origins of bacterial and archaeal viruses on sinking particles reaching the abyssal ocean. *ISME J.* **16**, 1627–1635 (2022). 35236926 10.1038/s41396-022-01202-1 PMC9122931

[20] Saunders, J. K. et al. Microbial functional diversity across biogeochemical provinces in the central Pacific Ocean. *Proc. Natl Acad. Sci. USA* **119**, e2200014119 (2022). 36067300 10.1073/pnas.2200014119 PMC9477243

[21] Bergauer, K. et al. Organic matter processing by microbial communities throughout the Atlantic water column as revealed by metaproteomics. *Proc. Natl Acad. Sci. USA* **115**, E400–E408 (2018). 29255014 10.1073/pnas.1708779115 PMC5776962

[22] Saito, M. A. et al. Multiple nutrient stresses at intersecting Pacific Ocean biomes detected by protein biomarkers. *Science* **345**, 1173–1177 (2014). 25190794 10.1126/science.1256450

[23] Kleiner, M. et al. Assessing species biomass contributions in microbial communities via metaproteomics. *Nat. Commun.* **8**, 1558 (2017). 29146960 10.1038/s41467-017-01544-x PMC5691128

[24] Sunagawa, S. et al. Ocean plankton. Structure and function of the global ocean microbiome. *Science* **348**, 1261359 (2015). 25999513 10.1126/science.1261359

[25] Salazar, G. et al. Gene expression changes and community turnover differentially shape the global ocean metatranscriptome. *Cell* **179**, 1068–1083 e1021 (2019). 31730850 10.1016/j.cell.2019.10.014 PMC6912165

[26] Acinas, S. G. et al. Deep ocean metagenomes provide insight into the metabolic architecture of bathypelagic microbial communities. *Commun. Biol.* **4**, 604 (2021). 34021239 10.1038/s42003-021-02112-2 PMC8139981

[27] Roux, S. et al. Ecogenomics and potential biogeochemical impacts of globally abundant ocean viruses. *Nature* **537**, 689–693 (2016). 27654921 10.1038/nature19366

[28] Blakeley-Ruiz, J. A. & Kleiner, M. Considerations for constructing a protein sequence database for metaproteomics. *Comput. Struct. Biotechnol. J.* **20**, 937–952 (2022). 35242286 10.1016/j.csbj.2022.01.018 PMC8861567

[29] Jagtap, P. et al. A two-step database search method improves sensitivity in peptide sequence matches for metaproteomics and proteogenomics studies. *Proteomics* **13**, 1352–1357 (2013). 23412978 10.1002/pmic.201200352 PMC3633484

[30] Carradec, Q. et al. A global ocean atlas of eukaryotic genes. *Nat. Commun.* **9**, 373 (2018). 29371626 10.1038/s41467-017-02342-1 PMC5785536

[31] Cohen, N. R. et al. Dinoflagellates alter their carbon and nutrient metabolic strategies across environmental gradients in the central Pacific Ocean. *Nat. Microbiol.* **6**, 173–186 (2021). 33398100 10.1038/s41564-020-00814-7

[32] Dong, H. P., Wang, D. Z., Dai, M., Chan, L. L. & Hong, H. S. Shotgun proteomics: tools for analysis of marine particulate proteins. *Limnol. Oceanogr. Methods* **7**, 865–874 (2009).

[33] Wang, D. Z., Dong, H. P., Xie, Z. X., Dai, M. H. & Hong, H. S. Metaproteomic characterization of dissolved organic matter in the water column of the South China Sea. *Limnol. Oceanogr.* **56**, 1641–1652 (2011).

[34] Sowell, S. M. et al. Transport functions dominate the SAR11 metaproteome at low-nutrient extremes in the Sargasso Sea.*ISME J.* **3**, 93–105 (2009). https://www.nature.com/articles/ismej200883#supplementary-information. 18769456 10.1038/ismej.2008.83

[35] Williams, T. J. et al. A metaproteomic assessment of winter and summer bacterioplankton from Antarctic Peninsula coastal surface waters. *ISME J.* **6**, 1883–1900 (2012). 22534610 10.1038/ismej.2012.28 PMC3446797

[36] Kanehisa, M. & Goto, S. KEGG: kyoto encyclopedia of genes and genomes. *Nucleic Acids Res.* **28**, 27–30 (2000). 10592173 10.1093/nar/28.1.27 PMC102409

[37] Bach, L. T. et al. The influence of plankton community structure on sinking velocity and remineralization rate of marine aggregates. *Glob. Biogeochem. Cycles* **33**, 971–994 (2019).

[38] Rocca, J. D. et al. Relationships between protein-encoding gene abundance and corresponding process are commonly assumed yet rarely observed. *ISME J.* **9**, 1693–1699 (2015). 25535936 10.1038/ismej.2014.252 PMC4511926

[39] DeLong, E. F. et al. Community genomics among stratified microbial assemblages in the ocean’s interior. *Science* **311**, 496–503 (2006). 16439655 10.1126/science.1120250

[40] Enke, T. N., Leventhal, G. E., Metzger, M., Saavedra, J. T. & Cordero, O. X. Microscale ecology regulates particulate organic matter turnover in model marine microbial communities. *Nat. Commun.* **9**, 2743 (2018). 30013041 10.1038/s41467-018-05159-8 PMC6048024

[41] Ho, T. K. In *Proc. 3rd international Conference on Document Analysis and Recognition* 278–282 (IEEE, 1995)

[42] Lindell, D. et al. Genome-wide expression dynamics of a marine virus and host reveal features of co-evolution. *Nature* **449**, 83–86 (2007). 17805294 10.1038/nature06130

[43] Stamieszkin, K., Steinberg, D. K. & Maas, A. E. Fecal pellet production by mesozooplankton in the subarctic Northeast Pacific Ocean. *Limnol. Oceanogr.* **66**, 2585–2597 (2021).

[44] De Corte, D. et al. Zooplankton‐derived dissolved organic matter composition and its bioavailability to natural prokaryotic communities. *Limnol. Oceanogr.*10.1002/lno.12272 (2022).

[45] Longhurst, A. R. & Harrison, W. G. Vertical nitrogen flux from the oceanic photic zone by diel migrant zooplankton and nekton. *Deep Sea Res. Part A. Oceanogr. Res. Pap.* **35**, 881–889 (1988).

[46] Cermak, N. et al. Direct single-cell biomass estimates for marine bacteria via Archimedes’ principle. *ISME J.* **11**, 825–828 (2017). 27922599 10.1038/ismej.2016.161 PMC5322313

[47] Simon, M. & Azam, F. Protein content and protein synthesis rates of planktonic marine bacteria. *Marine ecology progress series*. *Oldendorf* **51**, 201–213 (1989).

[48] Ducklow, H. Bacterial production and biomass in the oceans. *Microb. Ecol. oceans* **1**, 85–120 (2000).

[49] Almagro Armenteros, J. J. et al. SignalP 5.0 improves signal peptide predictions using deep neural networks. *Nat. Biotechnol.* **37**, 420–423 (2019). 30778233 10.1038/s41587-019-0036-z

[50] Vetter, Y. A., Deming, J. W., Jumars, P. A. & Krieger-Brockett, B. B. A predictive model of bacterial foraging by means of freely released extracellular enzymes. *Micro. Ecol.* **36**, 75–92 (1998). 10.1007/s002489900095 9622567

[51] Jiang, W. X. et al. A pathway for chitin oxidation in marine bacteria. *Nat. Commun.* **13**, 5899 (2022). 36202810 10.1038/s41467-022-33566-5 PMC9537276

[52] Diaz, J. M. et al. Widespread production of extracellular superoxide by heterotrophic bacteria. *Science* **340**, 1223–1226 (2013). 23641059 10.1126/science.1237331

[53] Herndl, G. J. et al. Contribution of Archaea to total prokaryotic production in the deep Atlantic Ocean. *Appl Environ. Microbiol.* **71**, 2303–2309 (2005). 15870315 10.1128/AEM.71.5.2303-2309.2005 PMC1087563

[54] Wei, W., Chen, X., Weinbauer, M. G., Jiao, N. & Zhang, R. Reduced bacterial mortality and enhanced viral productivity during sinking in the ocean. *ISME J.* **16**, 1668–1675 (2022). 35365738 10.1038/s41396-022-01224-9 PMC9123201

[55] Leu, A. O., Eppley, J. M., Burger, A. & DeLong, E. F. Diverse genomic traits differentiate sinking-particle-associated versus free-living microbes throughout the oligotrophic open ocean water column. *mBio* **13**, e0156922 (2022). 35862780 10.1128/mbio.01569-22 PMC9426571

[56] Sebastian, M. et al. High growth potential of long-term starved deep ocean opportunistic heterotrophic bacteria. *Front. Microbiol.* **10**, 760 (2019). 31024513 10.3389/fmicb.2019.00760 PMC6468046

[57] Winter, C., Bouvier, T., Weinbauer, M. G. & Thingstad, T. F. Trade-offs between competition and defense specialists among unicellular planktonic organisms: the “killing the winner” hypothesis revisited. *Microbiol. Mol. Biol. Rev.* **74**, 42–57 (2010). 20197498 10.1128/MMBR.00034-09 PMC2832346

[58] Lindell, D., Jaffe, J. D., Johnson, Z. I., Church, G. M. & Chisholm, S. W. Photosynthesis genes in marine viruses yield proteins during host infection. *Nature* **438**, 86–89 (2005). 16222247 10.1038/nature04111

[59] Coutinho, F. H. et al. Water mass age structures the auxiliary metabolic gene content of free-living and particle-attached deep ocean viral communities. *Microbiome* **11**, 118 (2023). 37237317 10.1186/s40168-023-01547-5 PMC10224230

[60] Walsh, D. & Mohr, I. Viral subversion of the host protein synthesis machinery. *Nat. Rev. Microbiol.* **9**, 860–875 (2011). 22002165 10.1038/nrmicro2655 PMC7097311

[61] Mizuno, C. M. et al. Numerous cultivated and uncultivated viruses encode ribosomal proteins. *Nat. Commun.* **10**, 752 (2019). 30765709 10.1038/s41467-019-08672-6 PMC6375957

[62] Sieradzki, E. T., Ignacio-Espinoza, J. C., Needham, D. M., Fichot, E. B. & Fuhrman, J. A. Dynamic marine viral infections and major contribution to photosynthetic processes shown by spatiotemporal picoplankton metatranscriptomes. *Nat. Commun.* **10**, 1169 (2019). 30862830 10.1038/s41467-019-09106-z PMC6414667

[63] Kieft, B. et al. Phytoplankton exudates and lysates support distinct microbial consortia with specialized metabolic and ecophysiological traits. *Proc. Natl Acad. Sci. USA* **118**, e2101178118 (2021). 34620710 10.1073/pnas.2101178118 PMC8521717

[64] Zimmerman, A. E., Podowski, J. C., Gallagher, G. E., Coleman, M. L. & Waldbauer, J. R. Tracking nitrogen allocation to proteome biosynthesis in a marine microbial community. *Nat. Microbiol.*10.1038/s41564-022-01303-9 (2023). 10.1038/s41564-022-01303-9 36635571

[65] Qin, Q. L. et al. Oxidation of trimethylamine to trimethylamine N-oxide facilitates high hydrostatic pressure tolerance in a generalist bacterial lineage. *Sci. Adv.* **7**, eabf9941 (2021). 33771875 10.1126/sciadv.abf9941 PMC7997507

[66] Saito, M. A. et al. Abundant nitrite-oxidizing metalloenzymes in the mesopelagic zone of the tropical Pacific Ocean. *Nat. Geosci.* **13**, 355–362 (2020).

[67] Zhang, Y. et al. Nitrifier adaptation to low energy flux controls inventory of reduced nitrogen in the dark ocean. *Proc. Natl Acad. Sci. USA* **117**, 4823–4830 (2020). 32071230 10.1073/pnas.1912367117 PMC7060736

[68] Kitzinger, K. et al. Single cell analyses reveal contrasting life strategies of the two main nitrifiers in the ocean. *Nat. Commun.* **11**, 767 (2020). 32034151 10.1038/s41467-020-14542-3 PMC7005884

[69] Wiltshire, K. H. & Lampert, W. Urea excretion by Daphnia: a colony inducing factor in Scenedesmus? *Limnol. Oceanogr.* **44**, 1894–1903 (1999).

[70] Konstantinidis, K. T., Braff, J., Karl, D. M. & DeLong, E. F. Comparative metagenomic analysis of a microbial community residing at a depth of 4,000 meters at station ALOHA in the North Pacific subtropical gyre. *Appl. Environ. Microbiol.* **75**, 5345–5355 (2009). 19542347 10.1128/AEM.00473-09 PMC2725473

[71] Kitzinger, K. et al. Cyanate and urea are substrates for nitrification by Thaumarchaeota in the marine environment. *Nat. Microbiol* **4**, 234–243 (2019). 30531977 10.1038/s41564-018-0316-2 PMC6825518

[72] Bianchi, D., Weber, T. S., Kiko, R. & Deutsch, C. Global niche of marine anaerobic metabolisms expanded by particle microenvironments. *Nat. Geosci.* **11**, 263-+ (2018).

[73] Wilhelm, S. W. & Suttle, C. A. Viruses and nutrient cycles in the sea. *BioScience* **49**, 781–788 (1999).

[74] Dittmar, T. et al. Enigmatic persistence of dissolved organic matter in the ocean. *Nat. Rev. Earth Environ.* **2**, 570–583 (2021).

[75] Mestre, M., Borrull, E., Sala, M. & Gasol, J. M. Patterns of bacterial diversity in the marine planktonic particulate matter continuum. *ISME J.* **11**, 999–1010 (2017). 28045454 10.1038/ismej.2016.166 PMC5364349

[76] Paul, J. H. Extraction of microbial DNA from aquatic sources: marine environments. *Mol. Microb. Ecol. Man.* **1**, 1–13 (1996).

[77] Wisniewski, J. R. Filter-aided sample preparation for proteome analysis. *Methods Mol. Biol.* **1841**, 3–10 (2018). 30259475 10.1007/978-1-4939-8695-8_1

[78] Perez-Riverol, Y. et al. The PRIDE database resources in 2022: a hub for mass spectrometry-based proteomics evidences. *Nucleic Acids Res.* **50**, D543–D552 (2022). 34723319 10.1093/nar/gkab1038 PMC8728295

[79] Li, D., Liu, C. M., Luo, R., Sadakane, K. & Lam, T. W. MEGAHIT: an ultra-fast single-node solution for large and complex metagenomics assembly via succinct de Bruijn graph. *Bioinformatics* **31**, 1674–1676 (2015). 25609793 10.1093/bioinformatics/btv033

[80] Hyatt, D. et al. Prodigal: prokaryotic gene recognition and translation initiation site identification. *BMC Bioinform.* **11**, 119 (2010). 10.1186/1471-2105-11-119 PMC2848648 20211023

[81] Li, W. & Godzik, A. Cd-hit: a fast program for clustering and comparing large sets of protein or nucleotide sequences. *Bioinformatics* **22**, 1658–1659 (2006). 16731699 10.1093/bioinformatics/btl158

[82] Logares, R. et al. Metagenomic 16S rDNA Illumina tags are a powerful alternative to amplicon sequencing to explore diversity and structure of microbial communities. *Environ. Microbiol.* **16**, 2659–2671 (2014). 24102695 10.1111/1462-2920.12250

[83] Kopylova, E., Noe, L. & Touzet, H. SortMeRNA: fast and accurate filtering of ribosomal RNAs in metatranscriptomic data. *Bioinformatics* **28**, 3211–3217 (2012). 23071270 10.1093/bioinformatics/bts611

[84] Hildebrand, F., Tadeo, R., Voigt, A. Y., Bork, P. & Raes, J. LotuS: an efficient and user-friendly OTU processing pipeline. *Microbiome* **2**, 30 (2014). 27367037 10.1186/2049-2618-2-30 PMC4179863

[85] Milanese, A. et al. Microbial abundance, activity and population genomic profiling with mOTUs2. *Nat. Commun.* **10**, 1014 (2019). 30833550 10.1038/s41467-019-08844-4 PMC6399450

[86] Eng, J. K., McCormack, A. L. & Yates, J. R. An approach to correlate tandem mass spectral data of peptides with amino acid sequences in a protein database. *J. Am. Soc. Mass Spectrom.* **5**, 976–989 (1994). 24226387 10.1016/1044-0305(94)80016-2

[87] Huerta-Cepas, J. et al. eggNOG 5.0: a hierarchical, functionally and phylogenetically annotated orthology resource based on 5090 organisms and 2502 viruses. *Nucleic Acids Res.* **47**, D309–D314 (2019). 30418610 10.1093/nar/gky1085 PMC6324079

[88] Mistry, J. et al. Pfam: the protein families database in 2021. *Nucleic Acids Res.* **49**, D412–D419 (2021). 33125078 10.1093/nar/gkaa913 PMC7779014

[89] Cantalapiedra, C. P., Hernández-Plaza, A., Letunic, I., Bork, P. & Huerta-Cepas, J. eggNOG-mapper v2: functional annotation, orthology assignments, and domain prediction at the metagenomic scale. *Mol. Biol. Evol.* **38**, 5825–5829 (2021). 34597405 10.1093/molbev/msab293 PMC8662613

[90] Buchfink, B., Xie, C. & Huson, D. H. Fast and sensitive protein alignment using DIAMOND. *Nat. Methods* **12**, 59–60 (2015). 25402007 10.1038/nmeth.3176

[91] Ortiz, M. et al. Multiple energy sources and metabolic strategies sustain microbial diversity in Antarctic desert soils. *Proc. Natl Acad. Sci. USA* **118**, e2025322118 (2021). 10.1073/pnas.2025322118 PMC8609440 34732568

[92] Katoh, K., Rozewicki, J. & Yamada, K. D. MAFFT online service: multiple sequence alignment, interactive sequence choice and visualization. *Brief. Bioinforma.* **20**, 1160–1166 (2017). 10.1093/bib/bbx108 PMC6781576 28968734

[93] Price, M. N., Dehal, P. S. & Arkin, A. P. FastTree: computing large minimum evolution trees with profiles instead of a distance matrix. *Mol. Biol. Evol.* **26**, 1641–1650 (2009). 19377059 10.1093/molbev/msp077 PMC2693737

[94] Letunic, I. & Bork, P. Interactive Tree of Life (iTOL) v6: recent updates to the phylogenetic tree display and annotation tool. *Nucleic Acids Res.* **52(W1)**, W78–W82 (2024). 10.1093/nar/gkae268 PMC11223838 38613393

[95] Zhang, Y., Wen, Z., Washburn, M. P. & Florens, L. Improving label-free quantitative proteomics strategies by distributing shared peptides and stabilizing variance. *Anal. Chem.* **87**, 4749–4756 (2015). 25839423 10.1021/ac504740p

[96] Eilers, H., Pernthaler, J., Glockner, F. O. & Amann, R. Culturability and in situ abundance of pelagic bacteria from the North Sea. *Appl. Environ. Microbiol.* **66**, 3044–3051 (2000). 10877804 10.1128/aem.66.7.3044-3051.2000 PMC92109

[97] Morris, R. M. et al. SAR11 clade dominates ocean surface bacterioplankton communities. *Nature* **420**, 806–810 (2002). 12490947 10.1038/nature01240

[98] Massana, R., Murray, A. E., Preston, C. M. & DeLong, E. F. Vertical distribution and phylogenetic characterization of marine planktonic *Archaea* in the Santa Barbara Channel. *Appl. Environ. Microbiol.* **63**, 50–56 (1997). 8979338 10.1128/aem.63.1.50-56.1997 PMC168301

[99] Teira, E., Reinthaler, T., Pernthaler, A., Pernthaler, J. & Herndl, G. J. Combining catalyzed reporter deposition-fluorescence in situ hybridization and microautoradiography to detect substrate utilization by Bacteria and Archaea in the deep ocean. *Appl. Environ. Microbiol.* **70**, 4411–4414 (2004). 15240332 10.1128/AEM.70.7.4411-4414.2004 PMC444763

[100] Suzuki, M. T., Sherr, E. B. & Sherr, B. F. DAPI direct counting underestimates bacterial abundances and average cell-size compared to AO direct counting. *Limnol. Oceanogr.* **38**, 1566–1570 (1993).

[101] Zeder, M., Kohler, E., Zeder, L. & Pernthaler, J. A novel algorithm for the determination of bacterial cell volumes that is unbiased by cell morphology. *Microsc Microanal.* **17**, 799–809 (2011). 21910938 10.1017/S1431927611012104

[102] Loferer-Krossbacher, M., Klima, J. & Psenner, R. Determination of bacterial cell dry mass by transmission electron microscopy and densitometric image analysis. *Appl. Environ. Microbiol.* **64**, 688–694 (1998). 9464409 10.1128/aem.64.2.688-694.1998 PMC106103

[103] Sintes, E. & Herndl, G. J. Quantifying substrate uptake by individual cells of marine bacterioplankton by catalyzed reporter deposition fluorescence in situ hybridization combined with microautoradiography. *Appl Environ. Microbiol.* **72**, 7022–7028 (2006). 16950912 10.1128/AEM.00763-06 PMC1636203

[104] Breiman, L. Random forests. *Mach. Learn.* **45**, 5–32 (2001).

[105] Oksanen, J. et al. Community ecology package. *R Package Version* **2**, 321–326 (2013).

[106] Wickham, H. ggplot2. *Wiley Interdiscip. Rev. Comput. Stat.* **3**, 180–185 (2011).

[107] Gu, Z., Gu, L., Eils, R., Schlesner, M. & Brors, B. circlize Implements and enhances circular visualization in R. *Bioinformatics* **30**, 2811–2812 (2014). 24930139 10.1093/bioinformatics/btu393

[108] Kolde, R. & Kolde, M. R. Package ‘pheatmap’. *R. Package* **1**, 790 (2015).

