# Supplementary material for: Metaproteomic analysis decodes trophic interactions of microorganisms in the dark ocean
Source: Nat Commun. 2024 Jul 30;15:6411. doi: 10.1038/s41467-024-50867-z (PMC11289388; doi:10.1038/s41467-024-50867-z)
Supplement: Supplementary file 1 — Supplementary Information [file 41467_2024_50867_MOESM1_ESM.pdf]

Supplementary Materials for

Metaproteomic analysis decodes the trophic interaction of microbes in the dark ocean

Zihao Zhao[1*], Chie Amano[1], Thomas Reinthaler[1], Federico Baltar[1, 2], Mónica V. Orellana[3, 4], Gerhard J. Herndl[1, 5, 6*]

[1] Department of Functional and Evolutionary Ecology, Bio-Oceanography and Marine Biology Unit, University of Vienna, Djerassiplatz 1, A-1030 Vienna, Austria
[2] Shanghai Engineering Research Center of Hadal Science and Technology, College of Marine Sciences, Shanghai Ocean University, Shanghai, China
[3] Polar Science Center, Applied Physics Laboratory, University of Washington, Seattle, WA 98195, USA
[4] Institute for Systems Biology, Seattle, WA 98109, USA
[5] NIOZ, Department of Marine Microbiology and Biogeochemistry, Royal Netherlands Institute for Sea Research, Utrecht University, Den Burg, The Netherlands
[6] Environmental & Climate Research Hub, University of Vienna, Althanstr. 14, A-1090 Vienna, Austria
*Correspondence to: zihao.zhao@univie.ac.at, gerhard.herndl@univie.ac.at

This file includes:
Figures S1 to S15
Tables S1 to S4
Other Supplementary Material for this manuscript includes the following:
Supplementary Data 1-12

Table S1 Protein databases composition of the two-step search.

	Eukaryotes	Prokaryotes	Virus
Step 1	79,577,878	105,585,296	1,501,235
Step 2	120,026	627,607	3,348

Table S2 Protein statistics in the different databases (DB).

	Eukaryotic DB	Prokaryotic DB	Viral DB
Proteins	75,783	156,883	1,884
Peptides	174,414	367,452	3,407
Unique peptides	108,966	232,340	2,212
Peptide-spectrum matches (PSMs)	1,872,594	3,381,369	20,190

Table S3 Protein classification at the super-kingdom level

	Archaea	Bacteria	Eukaryota	Viruses	Unclassified
Proteins	7,163	156,187	55,494	6,213	9,493
Peptides	13,581	403,638	102,903	10,592	14,559
Unique peptides	8,880	249,485	66,745	7,106	11,302
Peptide-spectrum matches (PSMs)	84,156	3,972,871	1,021,299	71,614	124,213

Table S4 Taxa included in algae, zooplankton and fungi identified based on eukaryotic proteins

	Phylum
Algae	Bacillariophyta, Streptophyta, Chlorophyta, Rhodophyta Prasinodermophyta, Haptista
Zooplankton	Arthropoda, Chordata, Ciliophora, Apicomplexa, Euglenozoa Rotifera, Cnidaria, Echinodermata, Nematoda, Mollusca Chaetognatha, Foraminifera, Discosea, Bryozoa, Platyhelminthes Heterolobosea, Cercozoa, Hemichordata, Evosea, Endomyxa Porifera, Annelida, Brachiopoda, Perkinsozoa, Microsporidia Ctenophora, Xenacoelomorpha, Sipuncula
Fungi	Ascomycota, Oomycota, Basidiomycota, Mucoromycota, Chytridiomycota

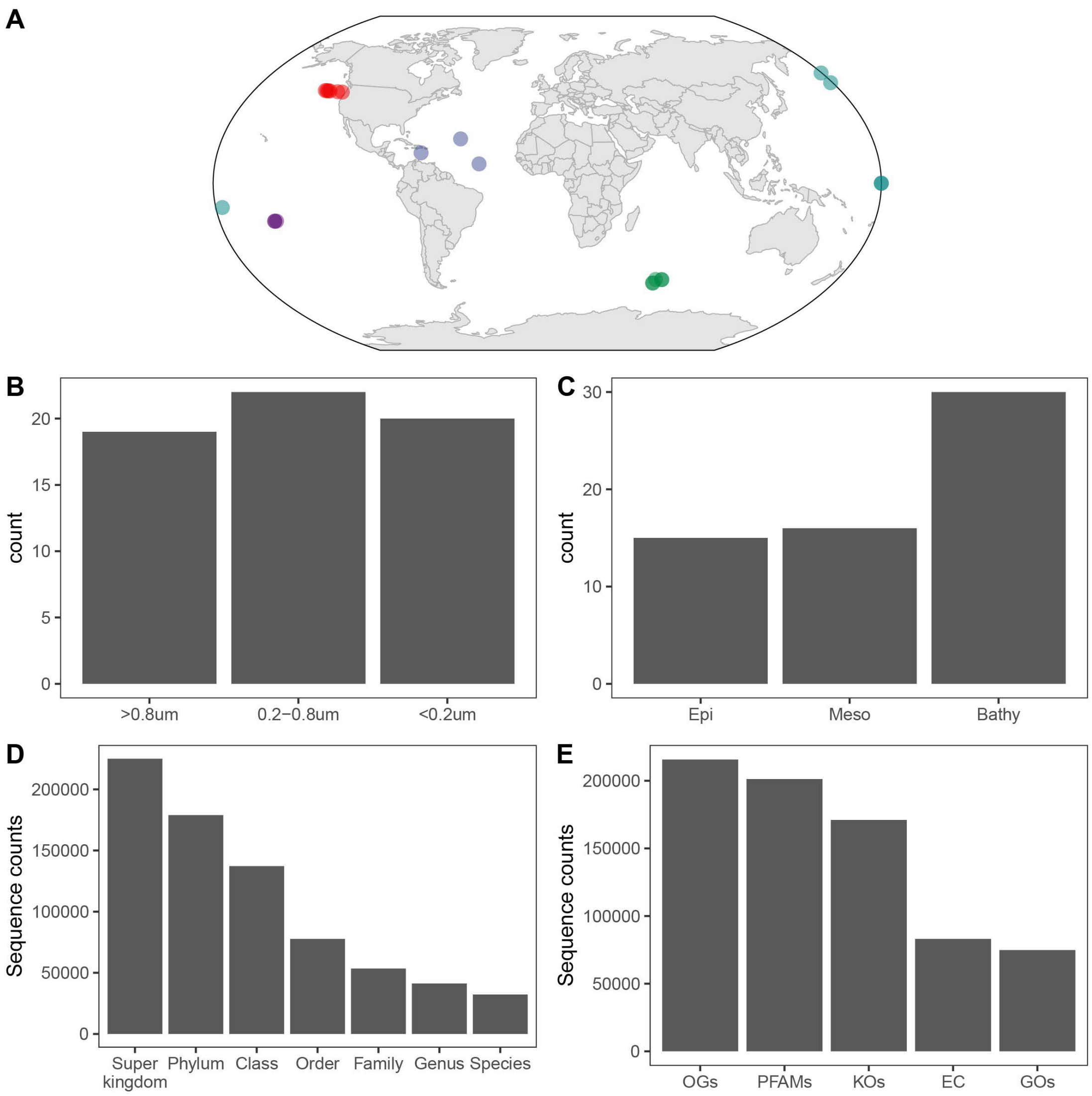

Fig. S1 Overview of metaproteomic samples. (**A**) Sampling map, the colors indicate different oceanic regions. Sample distribution in size-fractions (**B**) and depth layers (**C**). Taxonomic (**D**) and functional classification (**E**) of identified protein sequences. OGs, eggNOG orthology groups; PFAMs, protein families; KOs, KEGG-orthologues; EC, enzyme categories; GOs, Gene Ontologies. Epi, samples collected from epipelagic (<200m, n=15); Meso, samples collected from mesopelagic (200-1000m, n=16); Bathy, samples collected from bathypelagic (>1000m, n=30); >0.8μm, samples collected from the >0.8μm fraction (n=19); 0.2-0.8μm, samples collected from the 0.2-0.8μm fraction (n=22); <0.2μm, samples collected from the <0.2μm fraction (n=20).

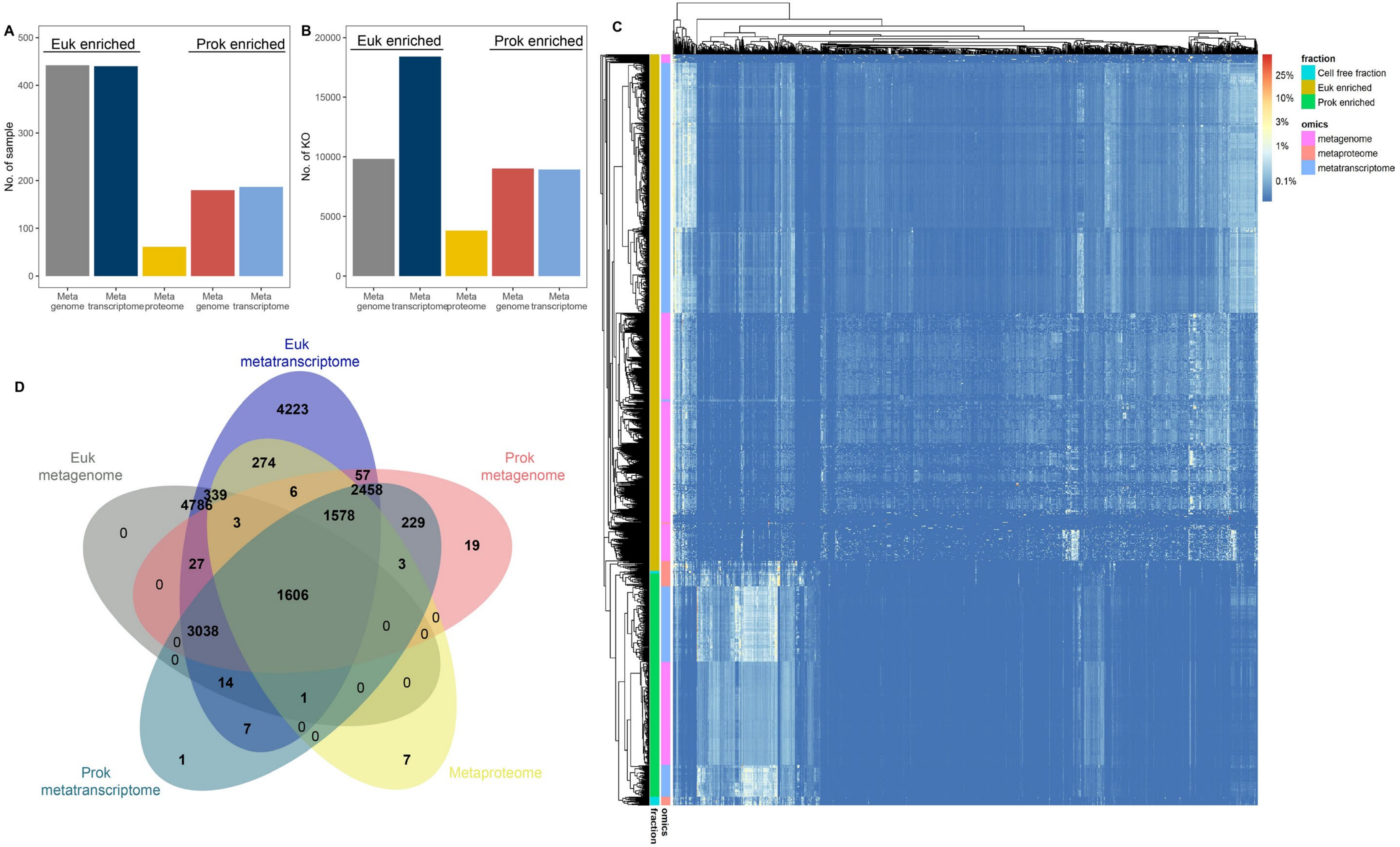

A
Euk enriched
Prok enriched
No. of sample
0
100
200
300
400
500
Meta genome
Meta transcriptome
Meta proteome
Meta genome
Meta transcriptome
B
Euk enriched
Prok enriched
No. of KO
0
5000
10000
15000
20000
Meta genome
Meta transcriptome
Meta proteome
Meta genome
Meta transcriptome
C
25%
10%
3%
1%
0.1%
fraction
Cell free fraction
Euk enriched
Prok enriched
omics
metagenome
metaproteome
metatranscriptome
fraction
omics
D
Euk metatranscriptome
Euk metagenome
Prok metagenome
Prok metatranscriptome
Metaproteome
4223
274
57
2458
339
4786
6
3
1578
229
0
19
27
0
1606
3
3038
0
0
0
0
0
14
1
0
0
7
0
0
1
7

Fig. S2 Profile of "omics" samples. (**A**) Number of -omics samples used for analyses. (**B**) Number of KOs detected in each omics group. (**C**) Heatmap showing the distribution of KOs (relative abundance >1%) in different omics samples. (**D**) Venn diagram showing the distribution of KOs (relative abundance >1%) shared by different omics samples. Euk-enriched, samples collected in the >0.8μm fraction (0.8-5μm, 5-20μm, 20-180μm and 180-2000μm). Prok-enriched, samples collected in the 0.2-3μm fraction. Cell-free fraction, samples collected in the <0.2μm fraction. Prok-metagenome, prokaryotic metagenome; Prok-metatranscriptome, prokaryotic metatranscriptome; Euk-metagenome, eukaryotic metagenome; Euk-metatranscriptome, eukaryotic metatranscriptome.

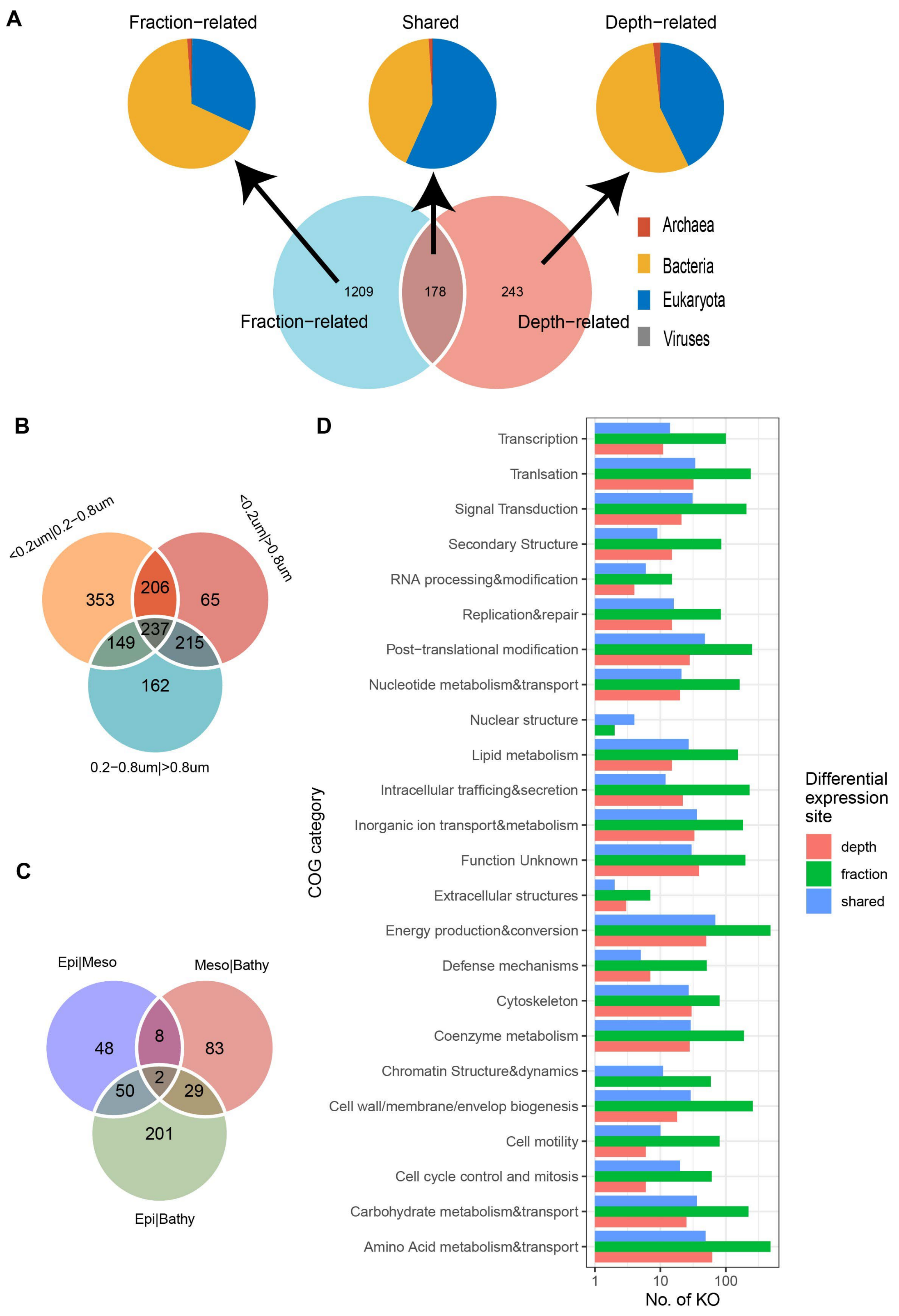
A
Fraction−related
Shared
Depth−related
1209
178
243
Fraction−related
Depth−related
Archaea
Bacteria
Eukaryota
Viruses
B
<0.2um|0.2−0.8um
<0.2um|>0.8um
0.2−0.8um|>0.8um
353
206
65
237
149
215
162
C
Epi|Meso
Meso|Bathy
Epi|Bathy
48
8
83
2
50
29
201
D
COG category
Transcription
Tranlsation
Signal Transduction
Secondary Structure
RNA processing&modification
Replication&repair
Post−translational modification
Nucleotide metabolism&transport
Nuclear structure
Lipid metabolism
Intracellular trafficing&secretion
Inorganic ion transport&metabolism
Function Unknown
Extracellular structures
Energy production&conversion
Defense mechanisms
Cytoskeleton
Coenzyme metabolism
Chromatin Structure&dynamics
Cell wall/membrane/envelop biogenesis
Cell motility
Cell cycle control and mitosis
Carbohydrate metabolism&transport
Amino Acid metabolism&transport
1
10
100
No. of KO
Differential expression site
depth
fraction
shared

Fig. S3 Profile of differentially expressed KOs in the metaproteome. (**A**) Comparison based on relative abundance of KO profiles of 1,630 identified KOs (Venn diagram) that may shape the clustering pattern of the metaproteome; the taxonomic origin of these KOs varied (pie chart). **(B)** Venn diagram showing the number of KOs differentially expressed between size fractions. **(C)** Venn diagram showing the number of KOs differentially expressed between depths. (**D**) Distribution of differentially expressed KOs in Clusters of Orthologous Genes (COG) category. Epi, samples collected from epipelagic (<200m, n=15); Meso, samples collected from mesopelagic (200-1000m, n=16); Bathy, samples collected from bathypelagic (>1000m, n=30); >0.8μm, samples collected from the >0.8μm fraction (n=19); 0.2-0.8μm, samples collected from the 0.2-0.8μm fraction (n=22); <0.2μm, samples collected from the <0.2μm fraction (n=20).

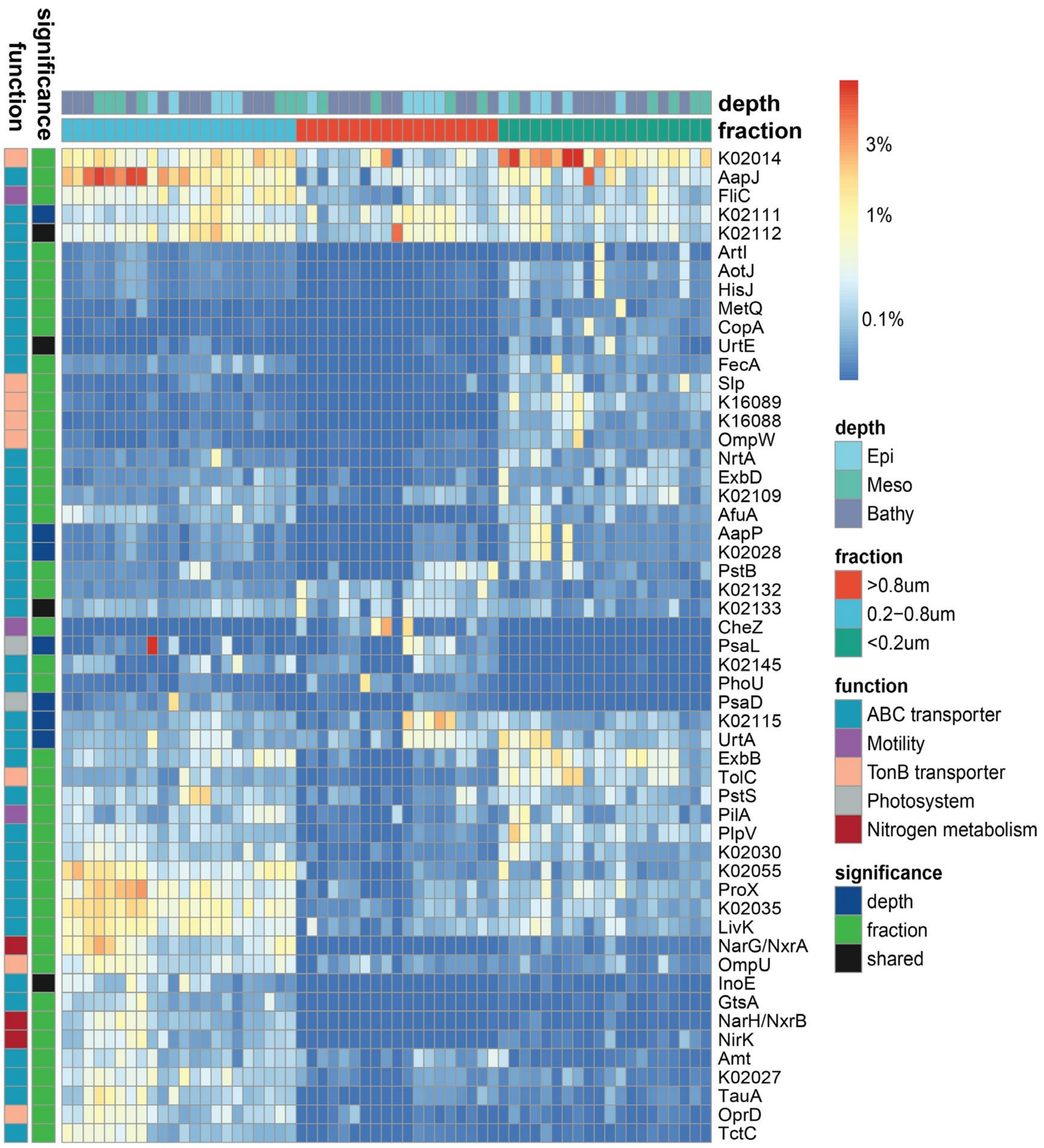

Fig. S4 Heatmap of KOs (relative abundance >1%) differentially expressed between fractions and depths. Epi, samples collected from epipelagic (<200m, n=15); Meso, samples collected from mesopelagic (200-1000m, n=16); Bathy, samples collected from bathypelagic (>1000m, n=30); >0.8µm, samples collected from the >0.8 µm fraction (n=19); 0.2-0.8µm, samples collected from the 0.2-0.8µm fraction (n=22); <0.2 µm, samples collected from the <0.2µm fraction (n=20). Depth, protein differentially abundant between depths layers; Fraction, protein differentially abundant between size fractions; Shared, protein differentially abundant between depth layers and size fractions.

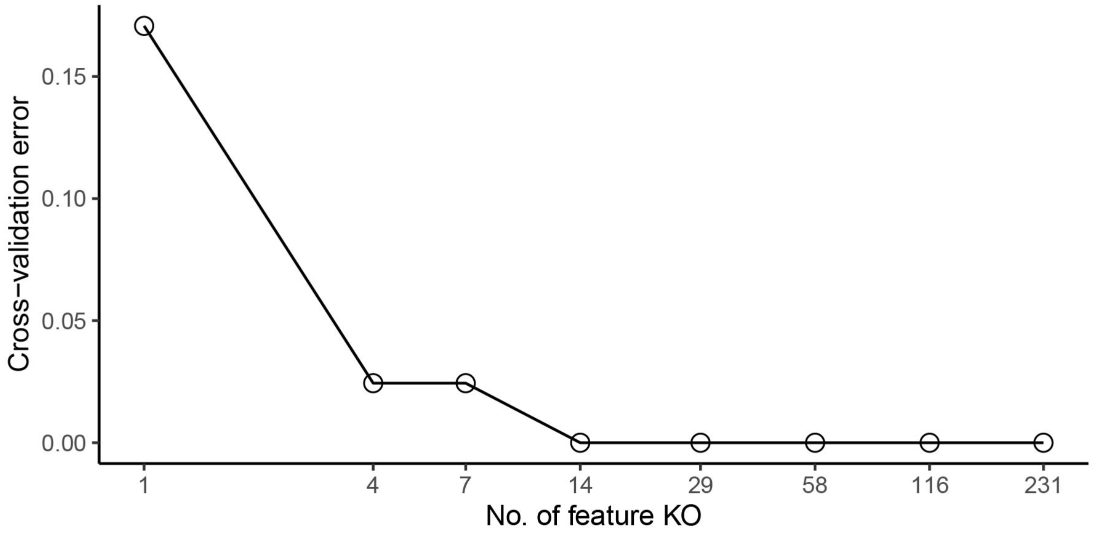

Fig. S5 Ten-fold cross-validation error as a function of the number of input KOs used to regress against the size-fraction in the metaproteomic dataset in order of variable importance.

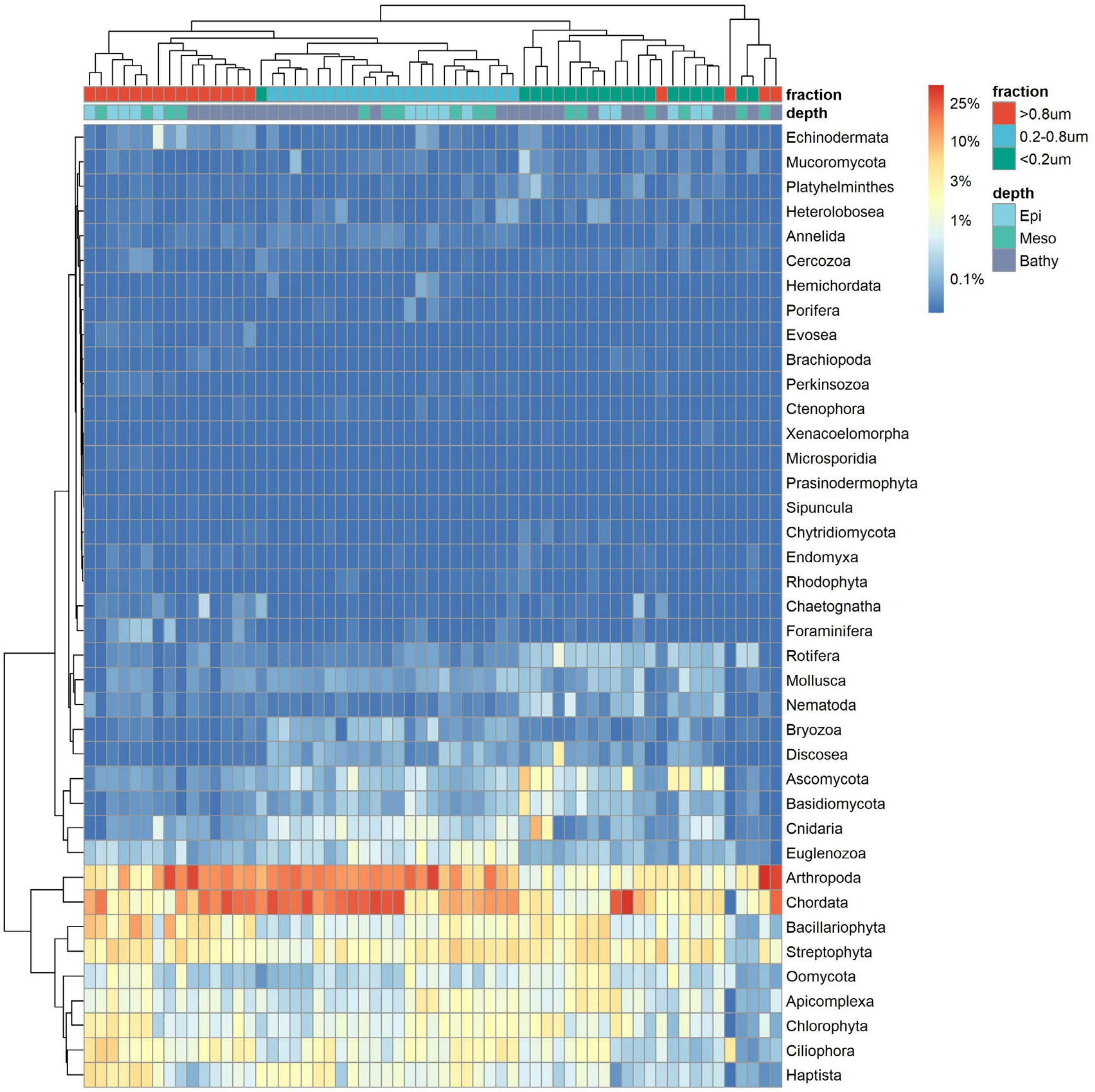

Fig. S6 Heatmap of the eukaryotic community composition (at the phylum level) in the metaproteomics dataset. Epi, samples collected from epipelagic (<200m, n=15); Meso, samples collected from mesopelagic (200-1000m, n=16); Bathy, samples collected from bathypelagic (>1000m, n=30); >0.8μm, samples collected from the >0.8 μm fraction (n=19); 0.2-0.8μm, samples collected from the 0.2-0.8μm fraction (n=22); <0.2 μm, samples collected from the <0.2μm fraction (n=20).

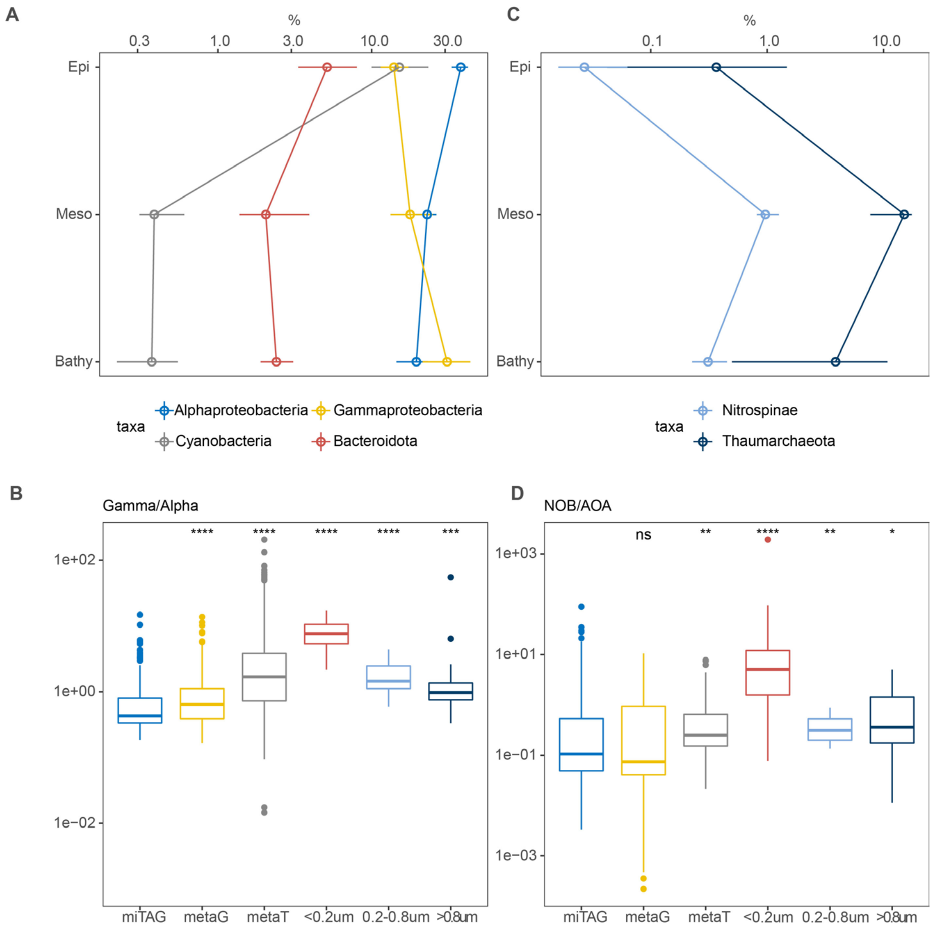

Fig. S7 Comparison between 16S rRNA gene, metagenome, metatranscriptome and metaproteome. (**A**) Depth profile of 16S rRNA genes (miTAG) for major prokaryotic taxonomic groups. (**B**) Ratio of Gammaproteobacteria/Alphaproteobacteria in the different 'omics' datasets as well as different size-fractions. (**C**) Depth profile of 16S rRNA gene (miTAG) for nitrifiers. (**D**) Ratio of Nitrospinae/Thaumarchaea (NOB/AOA) in the different 'omics' datasets as well as different size-fractions. Significance test (Wilcoxon test, two-side) was performed using 16S rRNA gene (miTAG) value as reference. *P<0.05, **P<0.01, ***P<0.001, ****P<0.0001, ns, not significant. metaG, mOTU data from metagenomes; metaT, mOTU data from metatranscriptomes. NOB, Nitrospina; AOA, Thaumarchaea. Epi, samples collected from epipelagic (<200m, n=15); Meso, samples collected from mesopelagic (200-1000m, n=16); Bathy, samples collected from bathypelagic (>1000m, n=30); >0.8µm, samples collected from the >0.8 µm fraction (n=19); 0.2-0.8µm, samples collected from the 0.2-0.8µm fraction (n=22); <0.2 µm, samples collected from the <0.2µm fraction (n=20). miTAG, 16S rRNA genes extracted from prokaryotic metagenome (n=345); metaG, mOTU derived from prokaryotic metagenome (n=345); metaT, mOTU derived from prokaryotic metatranscriptome (n=521). The points and ranges show the medians, the 25th and 75th IQR. Box shows median and interquartile range (IQR); whiskers show 1.5 × IQR of the lower and upper quartiles or range; outliers extend to the data range.

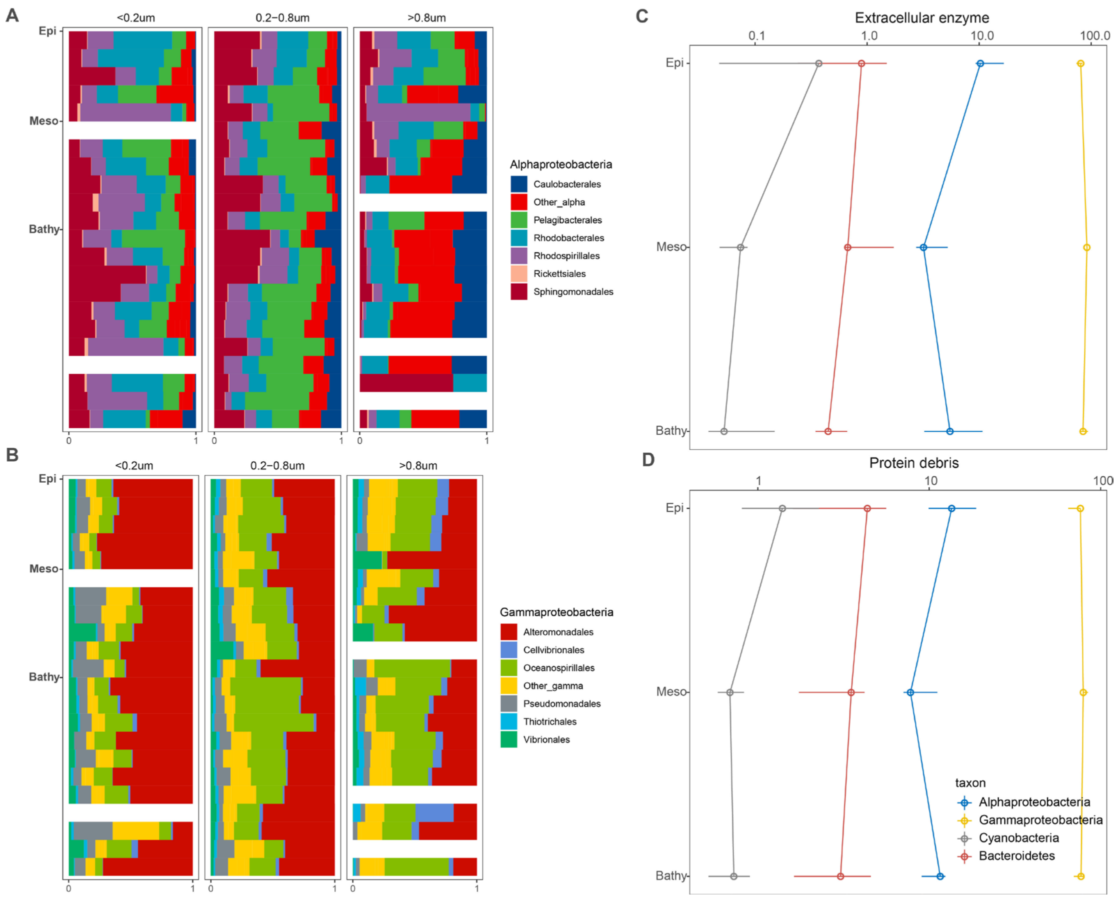

Fig. S8 Taxonomic profile of major bacterial proteins in each fraction. Taxonomic composition of Alphaproteobacteria (**A**) and Gammaproteobacteria (**B**) at the order level in the metaproteomic dataset. Contribution of major taxa to extracellular enzymes (**C**) and protein debris (**D**) in the <0.2μm fraction. Epi, samples collected from epipelagic (<200m, n=15); Meso, samples collected from mesopelagic (200-1000m, n=16); Bathy, samples collected from bathypelagic (>1000m, n=30); >0.8μm, samples collected from the >0.8 μm fraction (n=19); 0.2-0.8μm, samples collected from the 0.2-0.8μm fraction (n=22); <0.2 μm, samples collected from the <0.2μm fraction (n=20). The points and ranges show the medians, the 25th and 75th IQR.

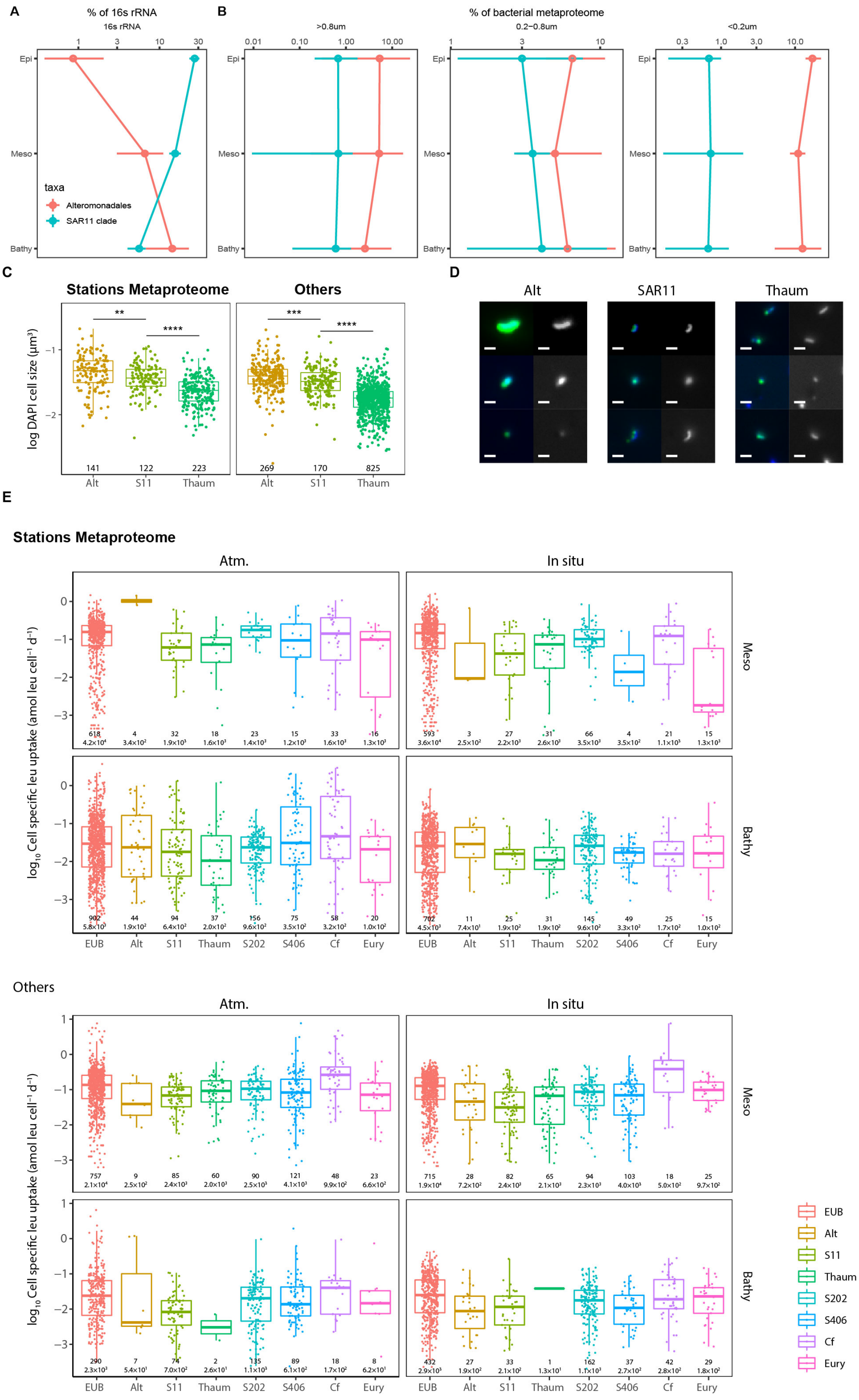

A
% of 16s rRNA
16s rRNA
B
% of bacterial metaproteome
>0.8um
0.2−0.8um
<0.2um
Epi
Meso
Bathy
taxa
Alteromonadales
SAR11 clade
C
Stations Metaproteome
Others
log DAPI cell size (µm³)
141
122
223
269
170
825
Alt
S11
Thaum
D
Alt
SAR11
Thaum
E
Stations Metaproteome
Atm.
In situ
Meso
Bathy
log₁₀ Cell specific leu uptake (amol leu cell⁻¹ d⁻¹)
EUB
Alt
S11
Thaum
S202
S406
Cf
Eury
Others
EUB
Alt
S11
Thaum
S202
S406
Cf
Eury

Fig. S9 Profile of Alteromonadales and SAR11 in the 16S rRNA gene (A) and metaproteomic dataset (B), DAPI-based cell size (C) and representative morphology (D) of CARD-FISH positive target organisms, and cell-specific leucine uptake rate (E). Sampling stations where metaproteome samples were collected are shown in separate panels (C, E; left side). Alt: *Alteromonas*/*Colwellia*, S11: SAR11 clade, Thaum: Thaumarchaeota, Bact: Bacteria. S202:
SAR202 clade, S406: SAR406 clade, Cf: Bacteroidetes, Eury: Euryarchaeota. Wilcoxon test (two side) was performed for each organism group; **P<0.01, ***P<0.001, ****P<0.0001. Numbers in the panels (**C**, **E**) indicate sample size of each boxplot. Numbers below the sample sizes in the panel (**E**) indicate abundance of active cells taking up leucine (cells/mL) in each target group. (**D**) shows microscopic images merging DAPI (blue) and FISH (green) channels. Scale bar: 1 µm. Epi, samples collected from epipelagic (<200m, n=15); Meso, samples collected from mesopelagic (200-1000m, n=16); Bathy, samples collected from bathypelagic (>1000m, n=30); >0.8µm, samples collected from the >0.8 µm fraction (n=19); 0.2-0.8µm, samples collected from the 0.2-0.8µm fraction (n=22); <0.2 µm, samples collected from the <0.2µm fraction (n=20). The points and ranges show the medians, the 25th and 75th IQR. Box shows median and interquartile range (IQR); whiskers show 1.5 × IQR of the lower and upper quartiles or range; outliers extend to the data range.

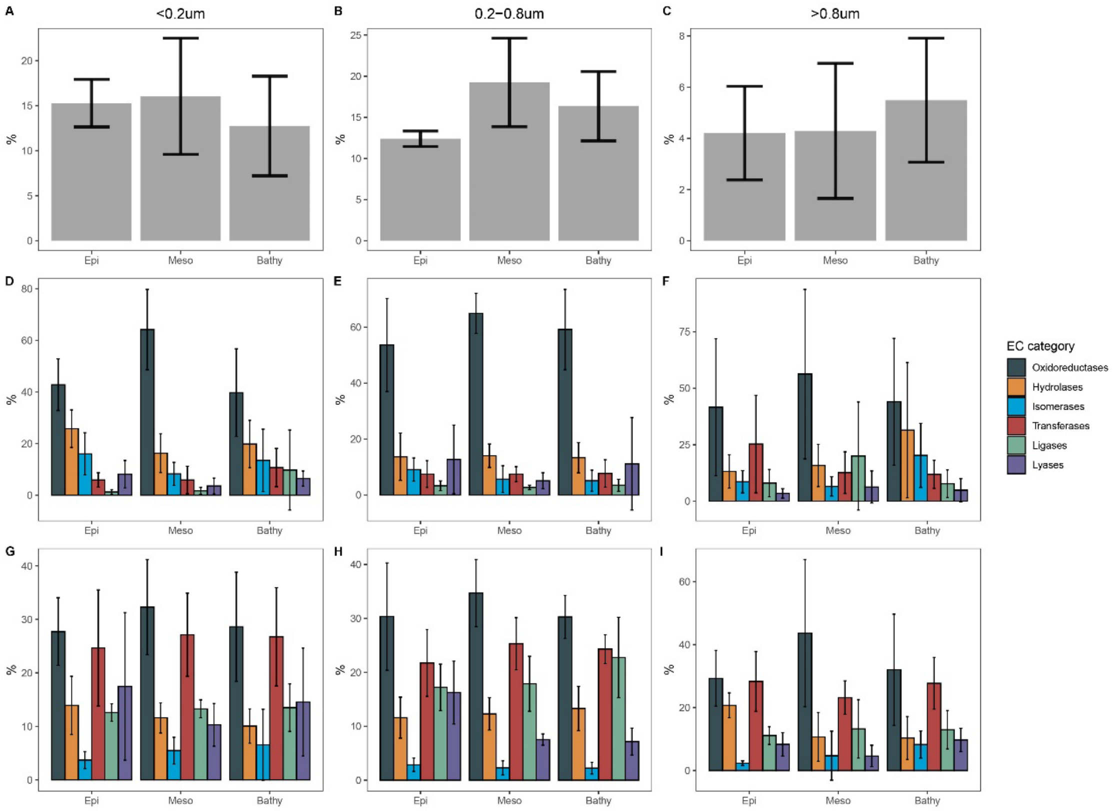

Fig. S10 Patterns of microbial extracellular enzymes in the metaproteomics dataset. (**A**-**C**) Depth profiles of the relative abundance of extracellular enzymes in the different size-fractions. (**D**-**F**) Functional categorization of extracellular enzymes. (**G**-**I**), Functional categorization of intracellular enzymes. Epi, samples collected from epipelagic (<200m, n=15); Meso, samples collected from mesopelagic (200-1000m, n=16); Bathy, samples collected from bathypelagic (>1000m, n=30); >0.8μm, samples collected from the >0.8 μm fraction (n=19); 0.2-0.8μm, samples collected from the 0.2-0.8μm fraction (n=22); <0.2 μm, samples collected from the <0.2μm fraction (n=20). Data are presented as mean values +/- SD

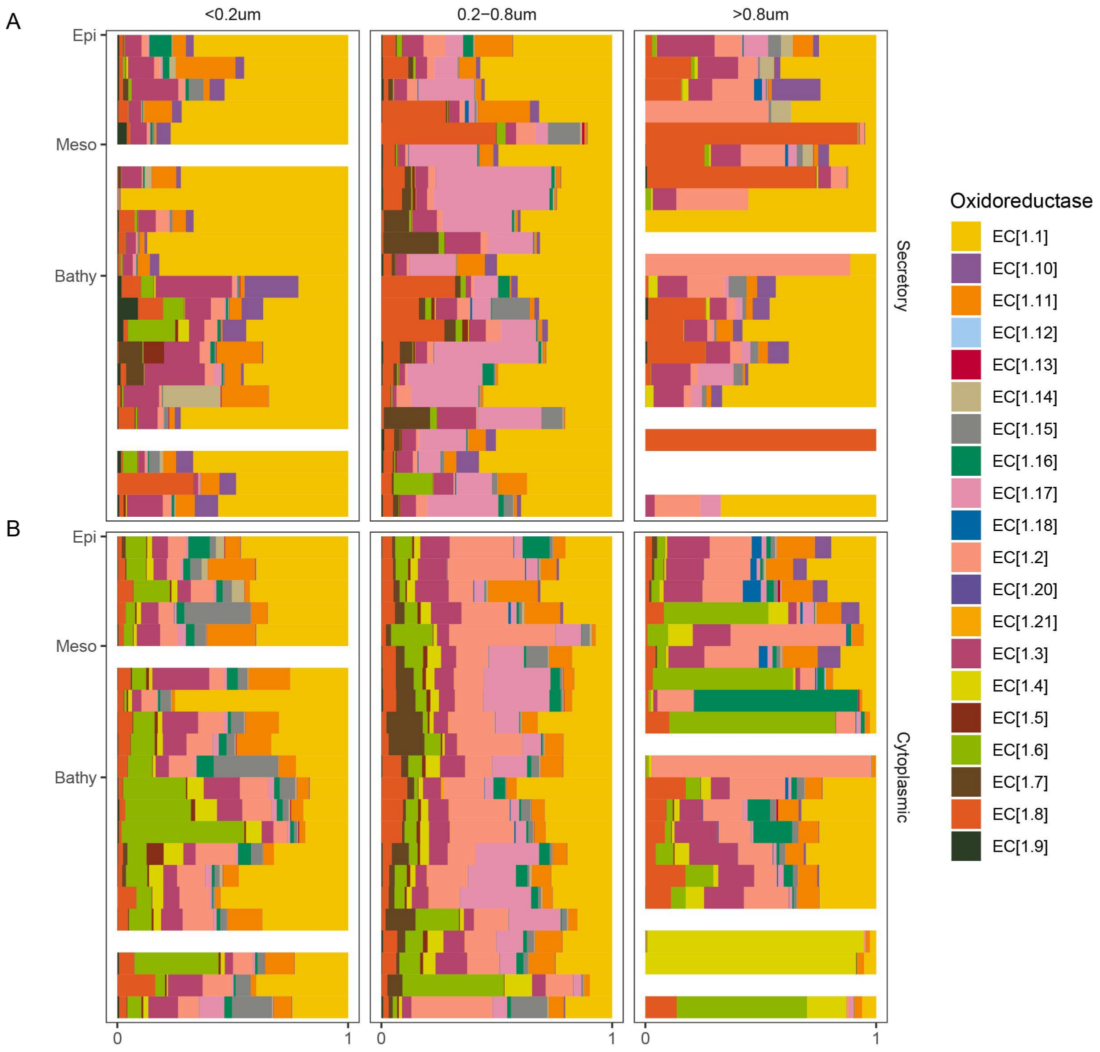

Fig. S11 Composition of secretory (A) and cytoplasmic (B) oxidoreductase in the metaproteomic dataset. Epi, samples collected from epipelagic (<200m, n=15); Meso, samples collected from mesopelagic (200-1000m, n=16); Bathy, samples collected from bathypelagic (>1000m, n=30); >0.8μm, samples collected from the >0.8 μm fraction (n=19); 0.2-0.8μm, samples collected from the 0.2-0.8μm fraction (n=22); <0.2 μm, samples collected from the <0.2μm fraction (n=20).

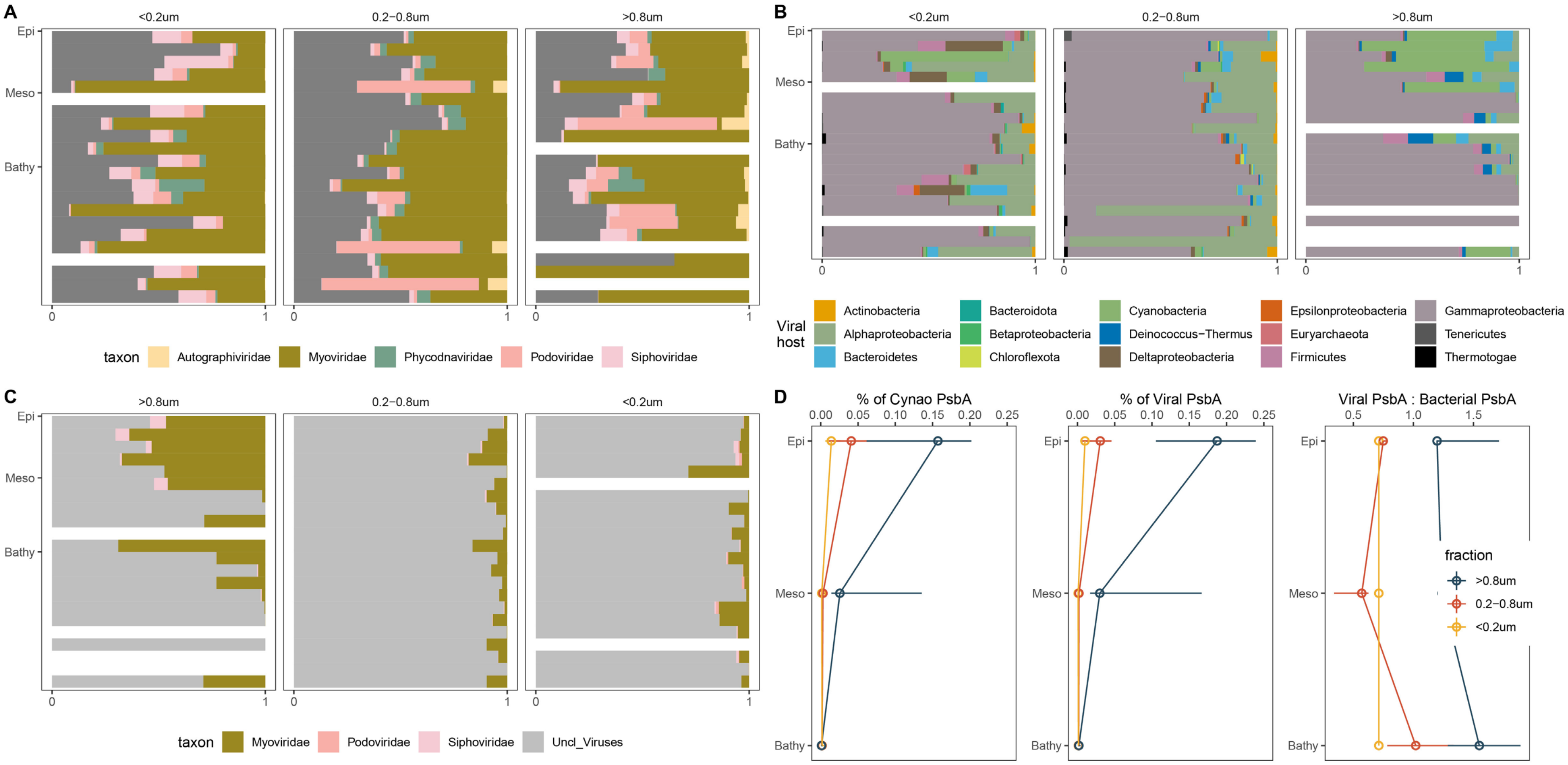
A
<0.2um
0.2−0.8um
>0.8um
Epi
Meso
Bathy
0
1
taxon
Autographiviridae
Myoviridae
Phycodnaviridae
Podoviridae
Siphoviridae
B
Viral host
Actinobacteria
Alphaproteobacteria
Bacteroidetes
Bacteroidota
Betaproteobacteria
Chloroflexota
Cyanobacteria
Deinococcus−Thermus
Deltaproteobacteria
Epsilonproteobacteria
Euryarchaeota
Firmicutes
Gammaproteobacteria
Tenericutes
Thermotogae
C
Uncl_Viruses
D
% of Cynao PsbA
% of Viral PsbA
Viral PsbA : Bacterial PsbA
0.00
0.05
0.10
0.15
0.20
0.25
0.5
1.0
1.5
fraction
>0.8um
0.2−0.8um
<0.2um

Fig. S12 Composition profiles of viruses. (**A**) Taxonomic composition of viruses at the family level in the metaproteomic dataset. (**B**) Taxonomic composition of putative hosts of viruses in the metaproteomic dataset. (C) Taxonomic composition of viruses putatively infecting Gammaproteobacteria. (**D**) Expression profiles of viral psbA protein and its relationship with cyanobacterial psbA. Epi, samples collected from epipelagic (<200m, n=15); Meso, samples collected from mesopelagic (200-1000m, n=16); Bathy, samples collected from bathypelagic (>1000m, n=30); >0.8μm, samples collected from the >0.8 μm fraction (n=19); 0.2-0.8μm, samples collected from the 0.2-0.8μm fraction (n=22); <0.2 μm, samples collected from the <0.2μm fraction (n=20). The points and ranges show the medians, the 25th and 75th IQR.

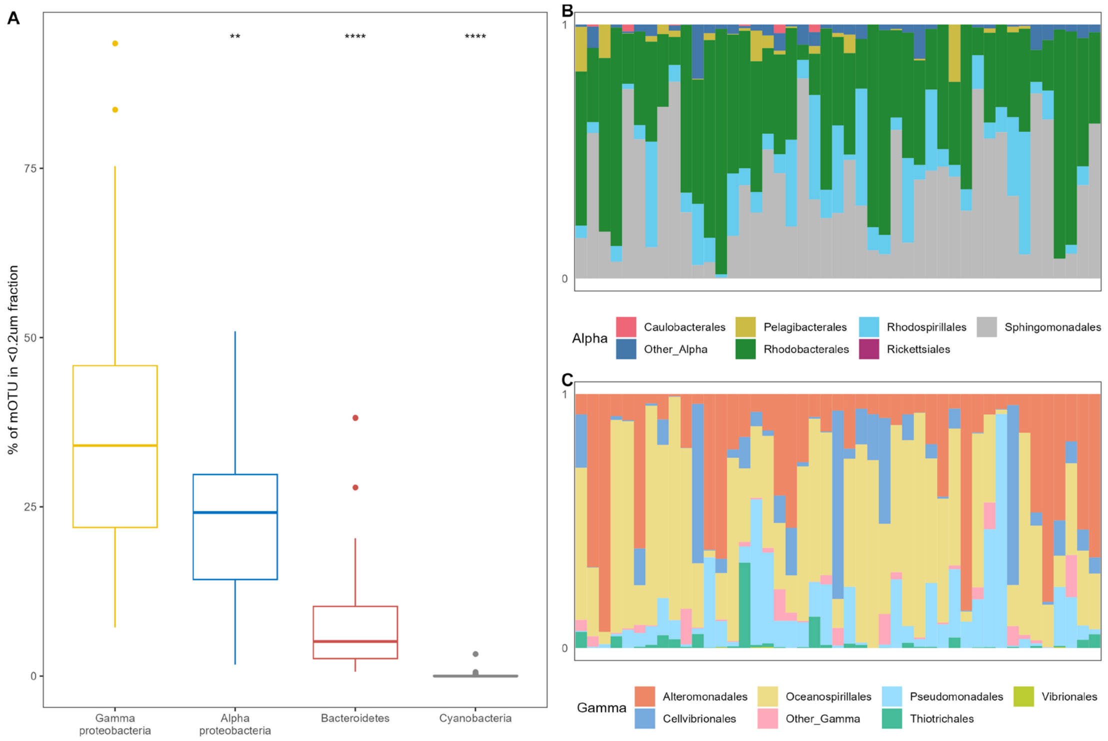

Fig. S13 Taxonomic profile (phylogenetic marker gene-based operational taxonomic units, mOTU) of metagenomes (n=45) collected in the <0.2μm size fraction. (**A**) Relative abundance of major taxonomic groups in metagenomes collected in the <0.2μm fraction. (**B-C**) Taxonomic profile of Alpha- and Gamma-proteobacteria at the order level in the metagenomes collected in the <0.2μm fraction, *x*-axis indicates metagenomic samples from <0.2μm (supplementary dataset2). Significance test (Wilcoxon test, two side) was performed using Gammaproteobacteria as reference. **P<0.01, ****P<0.0001. Box shows median and interquartile range (IQR); whiskers show 1.5 × IQR of the lower and upper quartiles or range; outliers extend to the data range.

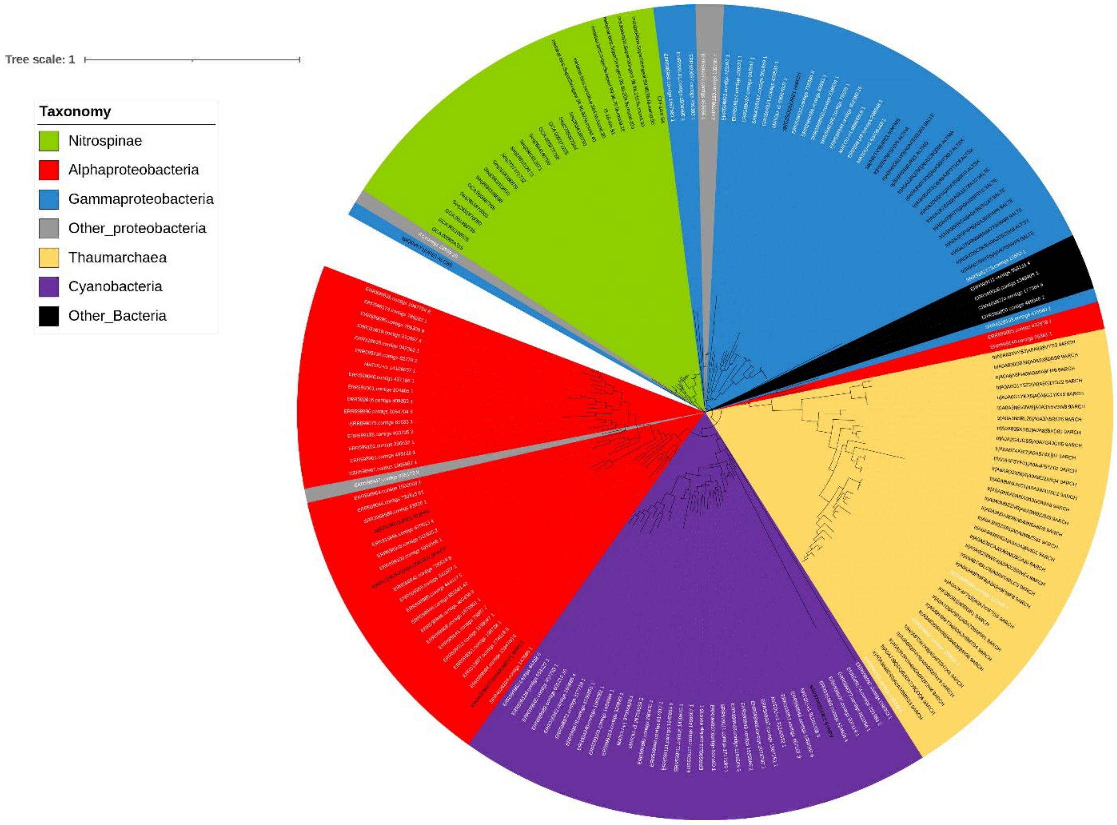

Fig. S14 Phylogenetic tree of UreC sequences. The color corresponds to the taxonomic annotation of the sequences. UreC sequences identified in our metaproteomic analysis are labeled in white and the sequences from the reference UreC are labeled in black.

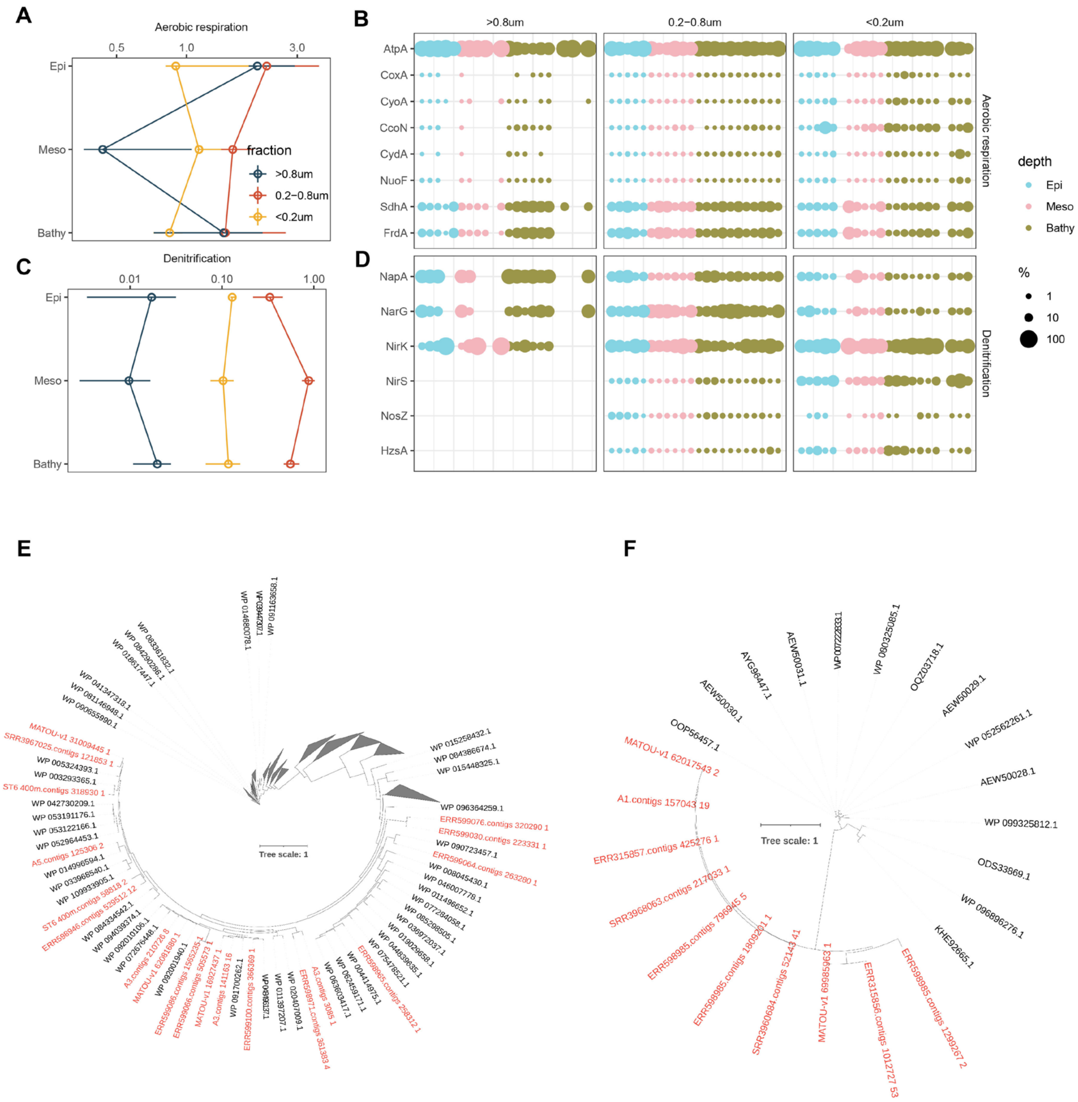

Fig. S15 Expression level of enzymes involved in aerobic respiration and denitrification. (**A**) Depth profile of enzymes involved in aerobic respiration. (**B**) Enzyme composition in aerobic respiration. (**C**) Depth profile of enzymes involved in denitrification. (**D**) Enzyme composition in denitrification. (**E-F**), phylogenetic placement of NosZ and HzsA identified in the metaproteome, protein sequences identified in the metaproteome are labelled in red. The relative abundance of each enzyme in **B** and **D** was normalized to the total enzyme pool in each process. AtpA, ATP synthase; CoxA/CyoA/CcoN/CydA, cytochrome oxidase; NuoF, NADH-quinone oxidoreductase subunit F; SdhA/FrdA, succinate dehydrogenase flavoprotein subunit; NapA/NarG, dissimilatory nitrate reductase; NirK/NirS, nitrite reductase; NosZ, nitrous-oxide reductase; HzsA, hydrazine synthase. Epi, samples collected from epipelagic (<200m, n=15); Meso, samples collected from mesopelagic (200-1000m, n=16); Bathy, samples collected from bathypelagic (>1000m, n=30); >0.8μm, samples collected from the >0.8 μm fraction (n=19); 0.2-0.8μm, samples collected from the 0.2-0.8μm fraction (n=22); <0.2 μm, samples collected from the <0.2μm fraction (n=20). The points and ranges show the medians, the 25th and 75th IQR.
