# Supplementary material for: Metaproteomic analysis decodes trophic interactions of microorganisms in the dark ocean
Source: Nat Commun. 2024 Jul 30;15:6411. doi: 10.1038/s41467-024-50867-z (PMC11289388; doi:10.1038/s41467-024-50867-z)
Supplement: Supplementary file 3 — Supplementary Data 1–12 [file 41467_2024_50867_MOESM3_ESM.zip › Caption of supplementary data.pdf]

Supplementary Data 1: Station information of metaproteomic samples Supplementary Data 2: Accession of metagenomics and metatranscriptomic reads for prokaryotic and eukaryotic community

Supplementary Data 3: Relative abundance (%) of KO in metaproteome

Supplementary Data 4: Relative abundance (%) of differentially expressed KO between size fractions or depth strata

Supplementary Data 5: Relative abundance (%) of superkingdoms Supplementary Data 6: Statistics of major eukaryotic groups in the metaproteome

Supplementary Data 7: Statistics of major prokaryotic groups in the metaproteome

Supplementary Data 8: Percentage of viral protein linked to putative hosts under different similarity thresholds

Supplementary Data 9: Statistics of major viruses infecting different bacterial hosts in the metaproteome

Supplementary Data 10: Statistics of cell specific leucine incorporation rate.

Supplementary Data 11: The accuracy, sensitivity, and specificity of the random forest model.

Supplementary Data 12: Classification rule for each decision tree.
