# Supplementary material for: Metaproteomic analysis decodes trophic interactions of microorganisms in the dark ocean
Source: Nat Commun. 2024 Jul 30;15:6411. doi: 10.1038/s41467-024-50867-z (PMC11289388; doi:10.1038/s41467-024-50867-z)
Supplement: Supplementary file 4 — Reporting Summary [file 41467_2024_50867_MOESM4_ESM.pdf]

nature portfolio

Corresponding author(s): Zihao Zhao, Gerhard J. Herndl

Last updated by author(s): Jul 9, 2024

Reporting Summary

Nature Portfolio wishes to improve the reproducibility of the work that we publish. This form provides structure for consistency and transparency in reporting. For further information on Nature Portfolio policies, see our Editorial Policies and the Editorial Policy Checklist.

Statistics

For all statistical analyses, confirm that the following items are present in the figure legend, table legend, main text, or Methods section.

n/a	Confirmed	
☐	☒	The exact sample size (n) for each experimental group/condition, given as a discrete number and unit of measurement
☒	☐	A statement on whether measurements were taken from distinct samples or whether the same sample was measured repeatedly
☐	☒	The statistical test(s) used AND whether they are one- or two-sided<br>*Only common tests should be described solely by name; describe more complex techniques in the Methods section.*
☒	☐	A description of all covariates tested
☒	☐	A description of any assumptions or corrections, such as tests of normality and adjustment for multiple comparisons
☐	☒	A full description of the statistical parameters including central tendency (e.g. means) or other basic estimates (e.g. regression coefficient) AND variation (e.g. standard deviation) or associated estimates of uncertainty (e.g. confidence intervals)
☐	☒	For null hypothesis testing, the test statistic (e.g. F, t, r) with confidence intervals, effect sizes, degrees of freedom and P value noted<br>*Give P values as exact values whenever suitable.*
☒	☐	For Bayesian analysis, information on the choice of priors and Markov chain Monte Carlo settings
☒	☐	For hierarchical and complex designs, identification of the appropriate level for tests and full reporting of outcomes
☒	☐	Estimates of effect sizes (e.g. Cohen's d, Pearson's r), indicating how they were calculated

Our web collection on statistics for biologists contains articles on many of the points above.

Software and code

Policy information about availability of computer code

Data collection	Proteome discover 2.1 (Thermo Fisher Scientific), ACMEtool3 (Zeder, M. 2005-2021, Software for Biology, http://www.technobiology.ch)
Data analysis	Megahit (v1.1.2), Prodigal (2.6.3) , CD-HIT (v4.6.8), SortMerRNA 4,mOTU 3.0, emapper 5.0, DIAMOND (v2.0.9), SignalP v5.0, R project (3.4.1), vegan (2.6), ggplot2 (3.5.1), randomforest (4.7), circlize (0.4.16), pheatmap (1.10.2),MAFFT (Version 7),FastTree (2.1), iTOL, (v6). R scripts for Permanova analysis, random forest analysis and different abundance analysis are available on github (https://github.com/zhaoz59/marine_metaP).

For manuscripts utilizing custom algorithms or software that are central to the research but not yet described in published literature, software must be made available to editors and reviewers. We strongly encourage code deposition in a community repository (e.g. GitHub). See the Nature Portfolio guidelines for submitting code & software for further information.

Data

Policy information about availability of data

All manuscripts must include a data availability statement. This statement should provide the following information, where applicable:

- Accession codes, unique identifiers, or web links for publicly available datasets
- A description of any restrictions on data availability
- For clinical datasets or third party data, please ensure that the statement adheres to our policy

Mass spectrum data are available via ProteomeXchange with identifier PXD034421(https://proteomecentral.proteomexchange.org/cgi/GetDataset?ID=PXD034421). Metagenomic reads have been deposited to the National Center for Biotechnology (NCBI) under Bioproject number PRJNA503889 (https://

www.ncbi.nlm.nih.gov/bioproject/PRJNA503889). The protein sequences used as metaproteomic database and metaproteomic results are available on FigureShare (https://doi.org/10.6084/m9.figshare.24570104.v1). Source data are also provided with this paper.

Human research participants

Policy information about studies involving human research participants and Sex and Gender in Research.

Reporting on sex and gender	*Use the terms sex (biological attribute) and gender (shaped by social and cultural circumstances) carefully in order to avoid confusing both terms. Indicate if findings apply to only one sex or gender; describe whether sex and gender were considered in study design whether sex and/or gender was determined based on self-reporting or assigned and methods used. Provide in the source data disaggregated sex and gender data where this information has been collected, and consent has been obtained for sharing of individual-level data; provide overall numbers in this Reporting Summary. Please state if this information has not been collected. Report sex- and gender-based analyses where performed, justify reasons for lack of sex- and gender-based analysis.*
Population characteristics	*Describe the covariate-relevant population characteristics of the human research participants (e.g. age, genotypic information, past and current diagnosis and treatment categories). If you filled out the behavioural & social sciences study design questions and have nothing to add here, write "See above."*
Recruitment	*Describe how participants were recruited. Outline any potential self-selection bias or other biases that may be present and how these are likely to impact results.*
Ethics oversight	*Identify the organization(s) that approved the study protocol.*

Note that full information on the approval of the study protocol must also be provided in the manuscript.

Field-specific reporting

Please select the one below that is the best fit for your research. If you are not sure, read the appropriate sections before making your selection.

☐ Life sciences ☐ Behavioural & social sciences ☒ Ecological, evolutionary & environmental sciences

For a reference copy of the document with all sections, see nature.com/documents/nr-reporting-summary-flat.pdf

Ecological, evolutionary & environmental sciences study design

All studies must disclose on these points even when the disclosure is negative.

Study description	Proteomics analysis of microbial proteins (including eukaryotes, bacteria, archaea and virus) collected from oceanic water columns (5-4000m) in major ocean basins (Pacific, Atlantic and Southern Ocean)
Research sample	Samples were size fractionated into three fractions: <0.2um, 0.2-0.8um and >0.8um. Eukaryotic detritus like algal aggregates, zooplankton carcasses and fecal pellets, cyanobacteria and particle-attached heterotrophic bacteria were mainly collected in the >0.8 um fraction and free-living prokaryotes in the 0.2-0.8 um fraction; viruses together with dissolved proteins/enzymes, either secreted or released, were obtained in the <0.2 um fraction
Sampling strategy	About 100-400 L of seawater were sequentially filtered through 0.8 um and 0.2 um pore-size polycarbonate membranes .The 0.2 um filtrate was further concentrated with tangential flow filtration driven by peristaltic pumps and using low protein binding membranes of a molecular weight cutoff at 5000 Da.
Data collection	Seawater was collected using Nisikin bottles. At each station where Nisikin bottles were deployed, a rosette CTD collected accompanying physiochemical parameters.
Timing and spatial scale	Samples collection was conducted during the research cruises: SO248 (BacGeoPac), M139 (MerMet 17-97), MOBYDICK, and OC1808C between 2016 to 2018. Detailed coordinates and depths for each sample can be found in supplementary dataset 1
Data exclusions	This study collected marine microbial proteins with cutoff of >5000 Dalton, proteins with molecular weight lower than 5000 Da was not collected due to technical limitations.
Reproducibility	Not applicable because samples were collected from highly dynamic oceanic regions
Randomization	Not applicable as sampling depths and sites were predetermined based on oceanographic features and maximal depth
Blinding	Not applicable as samples were analyzed associated with specific depth layers.

Did the study involve field work? ☒ Yes ☐ No

Field work, collection and transport

Field conditions	Samples were collected from oceanic water columns (5-4000m) in major ocean basins (Pacific, Atlantic and Southern Ocean)
Location	Detailed coordinates and depth for each sample can be found in supplementary dataset 1
Access & import/export	Samples were collected from international oceans, no permission needed for import
Disturbance	No disturbance caused from seawater sampling

Reporting for specific materials, systems and methods

We require information from authors about some types of materials, experimental systems and methods used in many studies. Here, indicate whether each material, system or method listed is relevant to your study. If you are not sure if a list item applies to your research, read the appropriate section before selecting a response.

Materials & experimental systems

n/a	Involved in the study
☒	☐ Antibodies
☒	☐ Eukaryotic cell lines
☒	☐ Palaeontology and archaeology
☒	☐ Animals and other organisms
☒	☐ Clinical data
☒	☐ Dual use research of concern

Methods

n/a	Involved in the study
☒	☐ ChIP-seq
☒	☐ Flow cytometry
☒	☐ MRI-based neuroimaging
